# Benzochromenopyrimidines: Synthesis, Antiproliferative Activity against Colorectal Cancer and Physicochemical Properties

**DOI:** 10.3390/molecules27227878

**Published:** 2022-11-15

**Authors:** Emna Choura, Fares Elghali, Paul J. Bernard, Dhouha Msalbi, José Marco-Contelles, Sami Aifa, Lhassane Ismaili, Fakher Chabchoub

**Affiliations:** 1 Laboratory of Applied Chemistry: Heterocycles, Lipids and Polymers, Faculty of Sciences of Sfax, University of Sfax, BP 802, Sfax 3000, Tunisia; 2 Laboratory of Molecular and Cellular Screening Processes, Centre of Biotechnology of Sfax, Sidi Mansour, Road Km 6, BP 1177, Sfax 3018, Tunisia; 3 Laboratoire LINC UR 481, Pôle de Chimie Médicinale, Université Franche-Comté, UFR Santé, 19, Rue Ambroise Paré, F-25000 Besançon, France; 4 Laboratory of Medicinal Chemistry (IQOG, CSIC), C/Juan de la Cierva 3, 28006 Madrid, Spain; 5 Center for Biomedical Network Research on Rare Diseases (CIBERER), CIBER, ISCIII, 28006 Madrid, Spain

**Keywords:** anticancer, anti-colorectal cancer, benzochromenopyrimidines

## Abstract

Ten new differently substituted 3-benzyl-5-aryl-3,5-dihydro-4*H*-benzo[6,7]chromeno[2,3-*d*]pyrimidin-4,6,11-triones **3** were synthesized by a simple and cost-efficient procedure in a one-pot, three-component reaction from readily available ethyl 2-amino-4-aryl-5,10-dioxo-5,10-dihydro-4*H*-benzo[*g*]chromene-3-carboxylates, benzylamine and triethyl orthoformate under solvent- and catalyst-free conditions. All the new compounds were screened for their antiproliferative activity against two colorectal-cancer-cell lines. The results showed that the compounds 3-benzyl-5-phenyl-3,5-dihydro-4*H*-benzo[6,7]chromeno[2,3-*d*]pyrimidine-4,6,11-trione (**3a**) and 3-benzyl-5-(3-hydroxyphenyl)-3,5-dihydro-4*H*-benzo[6,7]chromeno[2,3-*d*]pyrimidine-4,6,11-trione (**3g**) exhibited the most potent balanced inhibitory activity against human LoVo and HCT-116 cancer cells.

## 1. Introduction

Cancer is inherently a genetic disease. The accumulation of hereditary and/or acquired defects in genes that regulate cell proliferation and survival are responsible for the development of cancer [1]. Cancer is one of the leading causes of death worldwide, accounting for nearly 10 million deaths in 2020, or nearly one in six deaths [2]. The most common cancers are breast, lung, colorectal, and prostate. Colorectal cancer (CRC) is the most frequently diagnosed cancer in Europe and the US, and the second leading cause of cancer-related death [3].

Despite the availability of developed drugs, including targeted tumor therapies, the World Health Organization has announced that it is quite possible that the global burden of cancer will continue to increase in the coming years without real effective responses [4]. Therefore, the development of new anti-cancer drugs is a major goal and challenge for modern medicinal chemistry.

Benzo[*g*]chromenes bearing the naphthoquinone structural moiety occur in a variety of natural products that show a broad spectrum of biological activities [5,6,7,8]. On the other hand, several synthetic benzo[*g*]chromene derivatives have received growing interest in the pharmaceutical industry and in the study of organic synthesis due to their various biological and pharmacological properties, including antimicrobial [9,10], antileishmanial [11,12], and anticancer activities [13,14,15,16,17]. Pyrimidines and fused pyrimidines are also known to be preferred structures with diverse biological activities, and many of them are used as antimicrobial [18,19,20,21], anti-inflammatory [22,23], and anticancer therapeutic agents [24,25,26,27,28,29].

The association of these two scaffolds in a single molecule could create a synergistic effect in terms of activity and better drug-likeness properties.

This type of fused system has already been reported in the literature as an anti-Alzheimer’s [30,31], antioxidant [32], or antibacterial agent [33,34,35].

**Scheme 1:**
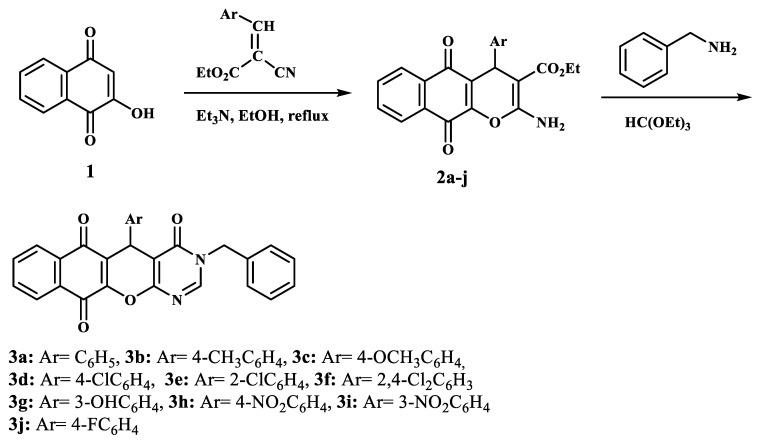
Synthesis of benzochromenopyrimidines **3a**–**j**.

In light of the above considerations, herein, we describe the synthesis of novel 3-benzyl-5-aryl-3,5-dihydro-4*H*-benzo[6,7]chromeno[2,3-*d*]pyrimidin-4,6,11-triones **3** (Scheme 1) bearing both biologically active benzo[*g*]chromene and pyrimidine functional motifs, by a simple and cost-effective procedure, in a one-pot, three-component reaction. All the synthesized products were evaluated as inhibitors of colon-cancer-cell proliferation.

## 2. Results

### 2.1. Synthesis

The synthesis of racemic benzochromenopyrimidine derivatives **3** was achieved by using a synthetic route shown in Scheme 1. In the first step, the preparation of the precursor ethyl 2-amino-4-aryl-5,10-dioxo-5,10-dihydro-4*H*-benzo[*g*]chromene-3-carboxylates was performed by mixing commercial 2-hydroxy-1,4-naphthoquinone **1** with ethyl 2-cyano-3-arylacrylates in the presence of a catalytic amount of triethylamine, at reflux [36,37,38,39]. Next, the one-pot condensation between compounds **2a**–**j**, benzylamine, and triethyl orthoformate, which were used as both reagent and solvent, at reflux, led to the corresponding 3-benzyl-5-aryl-3,5-dihydro-4*H*-benzo[6,7]chromeno[2,3-*d*]pyrimidine-4,6,11-triones **3a**–**j** (Table 1). The desired new compounds were obtained from modest-to-good yields (37–60%), and their analytical and spectroscopic data were in good agreement with their structures. In particular, the ^1^H NMR spectra showed the appearance of two doublets attributed to the methylene group protons –CH_2_–Ph between 4.95 and 5.15 ppm, with coupling constants for a typical AB system due to the magnetic non-equivalence of the methylene-group protons (see Supplementary Data).

Interestingly, we can note that the nature of the aryl group can have a significant impact on the yield of the reaction.

Indeed, a chlorine in position 2 of the aryl group decreased the yield from 59 to 43% compared to the absence of substituent. This effect was enhanced when the aryl group bore a chlorine in position 2 and another in position 4, where the yield decreased, this time to 37%.

On the other hand, no significant effect was observed when the different substituents were in position 3 or 4, including chlorine.

### 2.2. Biological Evaluation

#### 2.2.1. Cytotoxicity Test

The ten newly synthesized benzochromenopyrimidines **3a**–**j** were evaluated for their in vitro antiproliferative activity against two representative cell lines of human colon cancer, LoVo and HCT-116, using the standard 3-(4,5 dimethylthiazol-2-yl)-2,5-diphenyltetrazolium bromide (MTT) method. The cytotoxicity of each compound was assessed at different concentrations of 100 µM, 50 µM, 25 µM, 12.5 µM, 6.25 µM, 3.12 µM, 1.5 µM, 0.8 µM, and 0.4 µM. Oxaliplatin and 5-FU, the most common chemotherapy drugs used to treat colorectal cancer, were used as standards. The curve of the cell survival of LoVo and HCT-116 after treatment was obtained by the relation plotting of surviving fraction and drug concentration. All the results showed that the concentration of the cytotoxic compounds **3a**–**j** was inversely proportional to the percentage of cell viability. The value of concentration required to inhibit 50% of the cell viability (IC_50_) was determined and compared to those of the standard drugs, as shown in Table 2.

Several benzochromenopyrimidines showed good activities compared to the oxaliplatin and 5-FU against both types of cancer-cell line.

Concerning the LoVo cell lines, four compounds (**3a**: IC_50_ = 14.99 µM, **3b**: IC_50_ = 19.55 µM, **3e**: IC_50_ = 18.49 µM and **3g**: IC_50_ = 11.79 µM) showed activity that was better than or comparable to those of the two standards. In particular, compound **3g**, which showed an IC_50_ equal to 11.79 µM, was 1.7 times more active than the oxaliplatin and 5-FU. Thus, from the point of view of the structure–activity relationship (SAR), several conclusions can be drawn: (i) It is clear that the cytotoxic effect was related to the nature of the substituent in the aryl group. (ii) With the exception of compound **3b**, the substitution in position 4 of the aryl group did not enhance the activity. (iii) The two most promising compounds were the chlorine in position 2 and the hydroxy in position 3. This suggests that for better activity, a polar group in position 2 or 3 is necessary.

For the HCT-116 lines, six compounds showed better activity than the 5-FU (**3a**: IC_50_ = 15.92 µM, **3e**: IC_50_ = 13.70 µM, **3g**: IC_50_ = 13.61 µM, **3h**: IC_50_ = 16.45 µM, **3i**: IC_50_ = 7.15 µM and **3j**: IC_50_ = 23.85 µM), of which three (**3e**: IC_50_ = 13.70 µM, **3g**: IC_50_ = 13.61 µM and **3i**: IC_50_ = 7.15 µM) also showed activity that was better than or comparable to that of oxaliplatin (IC_50_ = 13.48 µM).

In this case, the SAR was also related to the nature of the aryl group. The comparison of the results of these compounds with oxaliplatin showed that the substitution in position 4 did not favor the activity. This unfavorable effect was related to the nature of the substituent (CH_3_ > OCH_3_ > Cl > F > NO_2_).

According to these results, the most balanced compounds were **3a** and **3g**, with an IC_50_ equal to 14.99 µM and 11.79 µM, respectively, against the Lovo cell lines, and 15.92µM and 13.61 µM, respectively, against the HCT-116 cell lines. Compounds **3a** and **3g** were, thus, more active than oxaliplatin and 5-FU against the LoVo cell lines and showed activities that were almost comparable to those of the oxaliplatin and two-fold more active than those of the 5-FU against the HCT-116 cell line.

#### 2.2.2. ADME Studies

Next, the physicochemical properties of the synthesized compounds were investigated by Data Warrior software, a chemical- and biological-data-visualization-and-analysis tool developed by Actelion/Idorsia Pharmaceuticals Ltd. (Table 3). This software utilizes different parameters of Lipinski’s rule of five (molecular weight, LogP, LogS, H-acceptors, H-donors, topological polar surface (TPSA) for the analysis of drug-like properties. All the compounds showed suitable MW values (MW < 500) for the pharmacokinetics of a drug in the human body with the exception of compound **3f**, which had a slightly higher value, 515.351 g/mol.

Lipophilicity is one of the properties of compounds that determine whether a molecule will cross the biological membrane, of which Log P (less than 5) is an important physiochemical example. Interestingly most of the compounds showed good lipophilicity, with Log P values between 3.3154 and 4.843. Only compound 3f, bearing two chlorines on the aromatic ring, showed a Log P higher than 5, with a value equal to 5.449. This value remained lower than 6.5, which is the upper limit for druggable compounds, indicating that **3f** was slightly lipophilic. Since lipophilicity plays a crucial role in determining the solubility of drug candidates in biological systems, we also calculated the Log S values of these compounds. All the compounds showed low aqueous solubility, suggesting reduced bioavailability. Structural modifications could be considered by introducing more polar groups to improve hydrophilicity and, therefore, duggability. Introducing an additional hydroxyl in position 2 of the aryl group of compound **3g** could be an option to enhance the solubility without a loss of activity. Indeed, the SAR showed that the presence of a polar group in 2 and 3 seems to be a necessary condition for the activity. Nevertheless, we can introduce polar groups, such as halogens or hydroxyls, on the aromatic rings of benzyl or benzochromene and study their impact on drugability and biological activity.

The number of donor and acceptor hydrogen bonds was also in agreement with Lipinski’s rule of five. Indeed, for all the compounds, the number of donor hydrogen bonds was lower than 5 and the number of acceptors was lower than 10. It can be noted, however, that compounds **3h** and **3i** had slightly more hydrogen acceptors than their analogues.

Data Warrior also calculates drug-likeness as a qualitative concept to predict whether synthesized compounds are drug-like. This parameter is calculated by using several data, such as LogP, LogS, and molar mass, as well as other parameters, such as the presence of structures with specific pharmacological properties (such as enones, which can be mutagenic and carcinogenic). It can be noted that all the compounds had an interesting drug-likeness prediction, except for compounds **3h** and **3i**, for which it was <0.

The TPSA corresponded to the Van der Waals surface of the molecules’ polar atoms (usually oxygen and nitrogen) and their attached hydrogens. The polar surface area was no greater than 140 Å^2^, as suggested by Veber’s Rule. Interestingly, all the compounds had a TPSA < 100 Å^2^, except for compounds **3h** and **3i**, for which it was 121.86 Å^2^.

## 3. Materials and Methods

Melting points (°C) were determined with a Kofler hot bench and were uncorrected. Analytical thin-layer chromatography (TLC) on silica-gel-precoated aluminum sheets (Type 60 F254, 0.25-mm thickness; from Merck, Darmstadt, Germany) was employed to follow the progress of the reactions and to check the purity and homogeneity of the synthesized products. Nuclear-magnetic-resonance spectra (NMR) were recorded on a Brucker DRX-400 Avance spectrometer (at 400 MHz for ^1^H and 100 MHz for ^13^C), using dimethylsulfoxide (DMSO-d_6_) as the solvent and tetramethylsilane (TMS) as internal standard. The chemical shifts are expressed in parts per million (ppm) and the multiplicities of ^1^H NMR signals were designated as follows: s: singlet; d: doublet; t: triplet; q: quartet; and m: multiplet. Coupling constants were expressed in hertz (Hz). High-resolution mass spectra (HRMS) were carried out by using a Bruker micrOTOF-Q II spectrometer (Bruker Daltonics) in positive electrospray ionization time-of-flight at UCA Clermont Ferrand, France.

### 3.1. Synthesis of Compounds ***2a**–**j***

#### 3.1.1. General Procedure for the Synthesis of Ethyl 2-Amino-4-(3-hydroxyphenyl)-5,10-dioxo-5,10-dihy dro-4*H*-benzo[*g*]chromene-3-carboxylate (**2a**–**j**)

An equimolar mixture of ethyl 2-cyano-3-arylacrylates and 2-hydroxy-1,4-naphthoquinone **1** was dissolved in 30 mL of ethanol in the presence of triethylamine as a catalyst. The reaction mixture was heated at reflux for 2 h. The resulting precipitate was collected by filtration and recrystallized from ethanol.

All compounds **2a**–**j** were previously described in the following studies: **2a**–**c** and **2i**–**j** [40], **2d** [37], **2e** [41], **2f** [38] and **2g** [42].

#### 3.1.2. General Procedure for the Synthesis of 3-Benzyl-5-aryl-3,5-dihydro-4H-benzo[6,7]chromeno[2,3-d]pyrimidin-4,6,11-triones (**3a**–**j**)

A mixture of ethyl 2-amino-4-aryl-5,10-dioxo-5,10-dihydro-4*H*-benzo[*g*]chromene-3-carboxylates **2a**–**j** (0.5 g, 1.33 mmol), benzylamine (0.14 g, 1.33 mmol), and triethyl orthoformate (3 mL) was heated under catalyst-free and solvent-free conditions. The completion of the reaction required a time of 3 to 5 h, as highlighted by TLC analysis, leading to the formation of a precipitate, which was collected by filtration, washed with ethanol, and dried. The obtained products **3a**–**j** were characterized by spectroscopic analysis (NMR and HRMS), which showed good agreement with the desired structure.

##### 3-Benzyl-5-phenyl-3,5-dihydro-4H-benzo[6,7]chromeno[2,3-d]pyrimidine-4,6,11-trione (**3a**)

Light-yellow solid; yield: 59%; mp > 260 °C; ^1^H NMR (400 MHz, DMSO-d_6_) δ 8.73 (s, 1H, CH_pyrimidine_), 8.10–8.07 (m, 1H), 7.93–7.91 (m, 1H), 7.89–7.85 (m, 2H), 7.38 (d, J = 7.4 Hz, 2H), 7.32–7.24 (m, 7H), 7.19–7.15 (m, 1H), 5.14 (d, J = 14.7 Hz, 1H, CH_2_), 5.13 (s, 1H, CH_pyran_), 4.98 (d, J = 14.7 Hz, 1H, CH_2_); ^13^C NMR (100 MHz, DMSO-d_6_) δ 183.0, 177.5, 160.4, 159.7, 152.2, 150.1, 142.4, 136.4, 135.1, 134.7, 131.5, 131.1, 129.1 (2C), 129.0 (2C), 128.7 (2C), 128.3, 128.2 (2C), 127.5, 126.6, 126.3, 123.3, 103.7, 49.8, 34.8. HRMS (ESI, M + H^+^) Calcd for C_28_H_19_N_2_O_4_: 447.1345. Found: 447.1339.

##### 3-Benzyl-5-(p-tolyl)-3,5-dihydro-4H-benzo[6,7]chromeno[2,3-d]pyrimidine-4,6,11-trione (**3b**)

Olive-green solid; yield: 53%; mp 252–4 °C; ^1^H NMR (400 MHz, DMSO-d_6_) δ 8.71 (s, 1H, CH_pyrimidine_), 8.09–8.07 (m, 1H), 7.90–7.86 (m, 3H), 7.30–7.24 (m, 7H), 7.05 (d, J = 7.1 Hz, 2H), 5.12 (d, J = 14.7 Hz, 1H, CH_2_), 5.08 (s, 1H, CH_pyran_), 4.98 (d, J = 14.7 Hz, 1H, CH_2_), 2.20 (s, 3H, CH_3_); ^13^C NMR (100 MHz, DMSO-d_6_) δ 183.0, 177.5, 160.4, 159.6, 152.1, 149.9, 139.5, 136.8, 136.4, 135.1, 134.7, 131.5, 131.0, 129.3 (2C), 129.1 (2C), 129.0 (2C), 128.3, 128.2 (2C), 126.6, 126.3, 123.5, 100.8, 49.8, 34.3, 21.0. HRMS (ESI, M + H^+^) Calcd for C_29_H_21_N_2_O_4_: 461.1501. Found: 461.1494.

##### 3-Benzyl-5-(4-methoxyphenyl)-3,5-dihydro-4H-benzo[6,7]chromeno[2,3-d]pyrimidine-4,6,11-trione (**3c**)

Ochre-yellow solid; yield: 60%; mp > 260 °C; ^1^H NMR (400 MHz, DMSO-d_6_) δ 8.71 (s, 1H, CH_pyrimidine_), 8.09–8.07 (m, 1H), 7.92–7.86 (m, 3H), 7.31 (m, 7H), 6.80 (d, J = 7.6 Hz, 2H), 5.12 (d, J = 14.2 Hz, 1H, CH_2_), 5.07 (s, 1H, CH_pyran_), 4.98 (d, J = 14.2 Hz, 1H, CH_2_), 3.68 (s, 3H, OCH_3_); ^13^C NMR (100 MHz, DMSO-d_6_) δ 183.1, 177.5, 160.4, 159.5, 158.7, 152.0, 149.8, 136.4, 135.1, 134.7, 134.6, 131.5, 131.1, 130.2 (2C), 129.1 (2C), 128.3, 128.2 (2C), 126.6, 126.3, 123.4, 114.1 (2C), 103.8, 55.4, 49.8, 33.9. HRMS (ESI, M + H^+^) Calcd for C_29_H_21_N_2_O_5_: 477.1450. Found: 477.1443.

##### 3-Benzyl-5-(4-chlorophenyl)-3,5-dihydro-4H-benzo[6,7]chromeno[2,3-d]pyrimidine-4,6,11-trione (**3d**)

Pale yellow solid; yield: 55%; mp > 260 °C; ^1^H NMR (400 MHz, DMSO-d_6_) δ 8.73 (s, 1H, CH_pyrimidine_), 8.08 (m, 1H), 7.90–7.86 (m, 3H), 7.42 (d, J = 6.7 Hz, 2H), 7.31 (m, 7H), 5.14–5.11 (m, 2H, CH_2_, CH_pyran_), 4.98 (d, J = 14.6 Hz, 1H, CH_2_); ^13^C NMR (100 MHz, DMSO-d_6_) δ 183.0, 177.4, 160.4, 159.6, 152.3, 150.2, 141.4, 136.4, 135.0, 134.7, 132.1 (2C), 131.4, 131.1 (2C), 129.0 (2C), 128.6 (2C), 128.3, 128.2 (2C), 126.6, 126.3, 122.6, 103.2, 49.8, 34.5. HRMS (ESI, M + H^+^) Calcd for C_28_H_18_^35^ClN_2_O_4_: 481.0955. Found: 481.0948.

##### 3-Benzyl-5-(2-chlorophenyl)-3,5-dihydro-4H-benzo[6,7]chromeno[2,3-d]pyrimidine-4,6,11-trione (**3e**)

Red solid brick; yield: 43%; mp > 260 °C; ^1^H NMR (400 MHz, DMSO-d_6_) δ 8.73 (s, 1H, CH_pyrimidine_), 8.08–8.06 (m, 1H), 7.88–7.84 (m, 3H), 7.43–7.41 (m, 2H), 7.34–7.18 (m, 7H), 5.48 (s, 1H, CH_pyran_), 5.10 (d, J = 14.6 Hz, 1H, CH_2_), 4.95 (d, J = 14.6 Hz, 1H, CH_2_); ^13^C NMR (100 MHz, DMSO-d_6_) δ 182.9, 177.5, 160.1, 159.8, 152.4, 150.2, 139.8, 136.4, 135.2, 134.7, 133.9, 132.7, 131.4, 130.8, 129.8 (2C), 129.1 (2C), 128.3, 128.0 (2C), 127.6, 126.5, 126.4, 122.5, 102.9, 49.7, 33.5. HRMS (ESI, M + H^+^) Calcd for C_28_H_18_^35^ClN_2_O_4_: 481.0955. Found: 481.0948.

##### 3-Benzyl-5-(2,4-dichlorophenyl)-3,5-dihydro-4H-benzo[6,7]chromeno[2,3-d]pyrimidine-4,6,11-trione (**3f**)

Ochre-yellow solid; yield: 37%; mp > 260 °C; ^1^H NMR (400 MHz, DMSO-d_6_) δ 8.74 (s, 1H, CH_pyrimidine_), 8.08–8.06 (m, 1H), 7.88–7.85 (m, 3H), 7.49–7.44 (m, 2H), 7.33–7.25 (m, 6H), 5.46 (s, 1H, CH_pyran_), 5.11 (d, J = 14.7 Hz, 1H, CH_2_), 4.96 (d, J = 14.7 Hz, 1H, CH_2_); ^13^C NMR (100 MHz, DMSO-d_6_) δ 183.0, 177.4, 160.1, 159.7, 152.4, 150.2, 139.1, 136.2, 135.3, 134.9, 134.8, 133.7, 132.7, 131.3, 130.7, 129.1 (2C), 129.0, 128.3, 128.0 (2C), 127.7, 126.6, 126.4, 122.0, 102.6, 49.7, 33.1. HRMS (ESI, M + H^+^) Calcd for C_28_H_17_^35^Cl_2_N_2_O_4_: 515.0565. Found: 515.0559.

##### 3-Benzyl-5-(3-hydroxyphenyl)-3,5-dihydro-4H-benzo[6,7]chromeno[2,3-d]pyrimidine-4,6,11-trione (**3g**)

Red solid brick; yield: 57%; mp 200–2 °C; ^1^H NMR (400 MHz, DMSO-d_6_) δ 9.37 (s, 1H, OH), 8.74 (s, 1H, CH_pyrimidine_), 8.07 (m, 1H), 7.93–7.85 (m, 3H), 7.34–7.31 (m, 5H), 7.05–7.02 (m, 1H), 6.80–6.76 (m, 2H), 6.56 (d, J = 7.9 Hz, 1H), 5.15 (d, J = 14.6 Hz, 1H, CH_2_), 5.04 (s, 1H, CH_pyran_), 5.00 (d, J = 14.6 Hz, 1H, CH_2_); ^13^C NMR (100 MHz, DMSO-d_6_) δ 183.0, 177.5, 160.4, 159.7, 157.6, 152.1, 149.9, 143.6, 136.4, 135.1, 134.7, 131.4, 131.0, 129.7, 129.1 (2C), 128.3, 128.2 (2C), 126.6, 126.4, 123.5, 119.7, 116.1, 114.6, 103.7, 49.8, 34.5. HRMS (ESI, M + H^+^) Calcd for C_28_H_19_N_2_O_5_: 463.1294. Found: 463.1286.

##### 3-Benzyl-5-(4-nitrophenyl)-3,5-dihydro-4H-benzo[6,7]chromeno[2,3-d]pyrimidine-4,6,11-trione (**3h**)

Ochre-yellow solid; yield: 50%; mp > 260 °C; ^1^H NMR (400 MHz, DMSO-d_6_) δ 8.73 (s, 1H, CH_pyrimidine_), 8.10–8.08 (m, 3H), 7.87–7.81 (m, 3H), 7.70 (d, J = 8.5 Hz, 2H), 7.32–7.27 (m, 5H), 5.21 (s, 1H, CH_pyran_), 5.09 (d, J = 14.7 Hz, 1H, CH_2_), 5.47 (d, J = 14.7 Hz, 1H, CH_2_); ^13^C NMR (100 MHz, DMSO-d_6_) δ 183.0, 177.3, 160.4, 159.7, 152.6, 150.4, 149.7, 146.9, 136.2, 135.1, 134.8, 131.3, 131.0, 130.8 (2C), 129.1 (2C), 128.4, 128.2 (2C), 126.6, 126.3, 123.7 (2C), 122.0, 102.6, 49.9, 35.3. HRMS (ESI, M + H^+^) Calcd for C_28_H_18_N_3_O_6_: 492.1196. Found: 492.1192.

##### 3-Benzyl-5-(3-nitrophenyl)-3,5-dihydro-4H-benzo[6,7]chromeno[2,3-d]pyrimidine-4,6,11-trione (**3i**)

Orange solid; yield: 52%; mp 258–260 °C; ^1^H NMR (400 MHz, DMSO-d_6_) δ 8.76 (s, 1H, CH_pyrimidine_), 8.24 (s, 1H), 8.06–8.04 (m, 2H), 7.89–7.85 (m, 4H), 7.56 (m, 1H), 7.29 (m, 5H), 5.25 (s, 1H, CH_pyran_), 5.12 (d, J = 14.7 Hz, 1H, CH_2_), 4.98 (d, J = 14.7 Hz, 1H, CH_2_); ^13^C NMR (100 MHz, DMSO-d_6_) δ 183.0, 177.3, 160.4, 159.7, 152.7, 150.5, 148.0, 144.4, 136.3, 136.1, 135.0, 134.7, 131.4, 131.2, 130.1, 129.0 (2C), 128.3, 128.2 (2C), 126.6, 126.3, 124.0, 122.6, 121.8, 102.8, 49.9, 35.2. HRMS (ESI, M + H^+^) Calcd for C_28_H_18_N_3_O_6_: 492.1196. Found: 492.1190.

##### 3-Benzyl-5-(4-fluorophenyl)-3,5-dihydro-4H-benzo[6,7]chromeno[2,3-d]pyrimidine-4,6,11-trione (**3j**)

Ochre-yellow solid, yield: 51%, mp > 260 °C; ^1^H NMR (400 MHz, DMSO-d_6_) δ 8.73 (s, 1H, CH_pyrimidine_), 8.09–8.07 (m, 1H), 7.93–7.83 (m, 3H), 7.45–7.42 (m, 2H), 7.33–7.26 (m, 5H), 7.09–7.05 (m, 2H), 5.13 (d, J = 14.7 Hz, 1H, CH_2_), 5.12 (s, 1H, CH_pyran_), 4.99 (d, J = 14.7 Hz, 1H, CH_2_); ^13^C NMR (100 MHz, DMSO-d_6_) δ 183.1, 177.4, 160.4, 159.6, 152.3, 150.1, 138.6, 136.4, 135.0, 134.7, 131.4 (2C), 131.2, 131.1, 129.0 (2C), 128.3, 128.2 (2C), 126.6, 126.3, 122.9, 115.5, 115.3, 103.5, 49.8, 34.2. HRMS (ESI, M + H^+^) Calcd for C_28_H_18_FN_2_O_4_: 465.1251. Found: 465.1244.

### 3.2. Biological Evaluation

#### 3.2.1. Materials and Methods

Two colon-cancer-cell lines (LoVo and HCT-116) were used in this study. Cells were cultured in RPMI-1640 media supplemented with 10% FBS and penicillin streptomycin. They were grown in a humidified incubator with 5% of CO_2_ at 37 °C.

#### 3.2.2. Cytotoxic Activity by MTT Assay

The synthesized compounds were solubilized in the DMSO as stock solutions (100 mM) and serial dilutions were prepared with cell-culture media just prior to use.

A 96-well plate was taken and seeded with 5000 cells/well, after which it was incubated overnight in an incubator at 37 °C with 5% of CO_2_. Next, the treatments were performed in triplicates and the plate was placed back in the incubator for 48h. After 48 h, the medium was removed and 100 µL of MTT was added into each well and incubated for 24 h. Subsequently, the MTT containing medium was removed from the wells. A total of 100 µL of SDS 10% was added into each well to dissolve the formazan crystals from the cells. Next, the plate was analyzed on micro plate reader (Varioskan Thermo Fisher) after 4 h. The absorbance was measured for each well with a wavelength of 570 nm. The IC_50_ values were then calculated.

### 3.3. Statistical Data Analyses

All experiments were performed in triplicate. Data were exposed as mean ± SD. Statistical analyses were performed by Student’s test. The normality and Leven’s test for homogeneity of variances were applied prior to one-way analysis of variance (ANOVA) and multiple mean comparisons were performed with Duncan’s test at *p* values ≤ 0.05 to investigate the significance differences in factors between synthesized compounds and standards (oxaliplatin and 5-FU) at a confidence level of 95%.

## 4. Conclusions

In the present study, a new series of benzochromenopyrimidine derivatives **3** was synthesized in a single step, by reacting ethyl 2-amino-4-aryl-5,10-dioxo-5,10-dihydro-4*H*-benzo[g]chromene-3-carboxylates **2** with benzylamine and triethyl orthoformate, both of which were readily available, without a solvent or catalyst.

The evaluation of the newly synthesized compounds for antitumor activity against the human-colon-cancer-cell lines LoVo and HCT-116 exhibited good results. Among the tested compounds, 3-benzyl-5-(3-hydroxyphenyl)-3,5-dihydro-4*H*-benzo[6,7]chromeno[2,3-*d*]pyrimidine-4,6,11-trione (**3g**) showed strong activity against the LoVo cell line with an IC_50_ value equal to 11.79 μM, and 3-benzyl-5-phenyl-3,5-dihydro-4*H*-benzo[6,7]chromeno[2,3-*d*]pyrimidine-4,6,11-trione (**3a**) also exhibited high antitumor activity against towards the LoVo cell line, with an IC_50_ value of 14.99 μM, comparing very well with standards, oxaliplatin and 5-FU. In addition, ligands **3a** and **3g** showed good activities against the HCT-116 cell line, with IC_50_ equal to 15.92 μM and 13.61 μM, respectively.

Interestingly, both compounds showed suitable physicochemical properties according to the drug-likeness score for druggability predicted by the Data Warrior software.

In summary, this preliminary study revealed that compounds **3a** and **3g** may be promising agents for further research into the treatment of colon cancer. It should be noted that products **3a** and **3g** were obtained in racemic form and that their antiproliferative activities could be attributed to one of the enantiomers.

Therefore, work is currently underway in our laboratories to develop analogues with better pharmacological profiles by identifying the contribution of each enantiomer to the biological activity. The results will be reported in due course.

## Figures and Tables

**Table 1:** Synthesis of benzo[6,7]chromeno[2,3-*d*]pyrimidine derivatives under one-pot solvent- and catalyst-free conditions.

Entry	Product	Ar	Time (h)	Yield (%)
1	3a	C_6_H_5_	3	59
2	3b	4-CH_3_C_6_H_4_	4	53
3	3c	4-OCH_3_C_6_H_4_	3	60
4	3d	4-ClC_6_H_4_	4	55
5	3e	2-ClC_6_H_4_	5	43
6	3f	2,4-Cl_2_C_6_H_3_	5	37
7	3g	3-OHC_6_H_4_	3	57
8	3h	4-NO_2_C_6_H_4_	5	50
9	3i	3-NO_2_C_6_H_4_	4	52
10	3j	4-FC_6_H_4_	3	51

**Table 2:** Results of in vitro cytotoxic activity of the synthesized compounds on human-colon-cancer-cell lines (LoVo and HCT-116) ^a^.

	IC_50_ (µM)	
Compounds	LoVo	HCT-116
**3a**	^#^ 14.99 ± 0.59 *	^#^ 15.92 ± 0.30
**3b**	19.55 ± 3.17	53.93 ± 7.52
**3c**	24.35 ± 3.30	40.32 ± 3.28
**3d**	30.11 ± 3.16	42.65 ± 3.41
**3e**	18.49 ± 0.57	^#^ 13.70 ± 0.74
**3f**	39.32 ± 5.13	56.03 ± 0.94
**3g**	^#^ 11.79 ± 0.16 *	^#^ 13.61 ± 2.51
**3h**	47.12 ± 3.83	16.45 ± 2.32
**3i**	33.14 ± 2.18	^#^ 7.15 ± 0.67 *
**3j**	49.03 ± 2.19	23.85 ± 3.08
**Oxaliplatin**	19.51 ± 2.35	13.48 ± 1.78
**5-FU**	19.99 ± 3.21	26.98 ± 1.87

^a^ The results are expressed as the mean number of IC50 ± SD (n = 3), (*) and (^#^) indicates a significant difference between standards (oxaliplatin and 5-FU respectively) and the synthesized compounds (at *p* ≤ 0.05, ANOVA; post hoc Dunnett test).

**Table 3:** Physicochemical properties of the synthesized compounds calculated by Data Warrior.

Name	Molweight (g/mol)	CLogP	CLogS	H-Donors	H-Acceptors	Lipinsky Violations	Drug-Likeness	TPSA (Å^2^)
**3a**	446.461	4.237	−7.271	0	6	0	4.8734	76.04
**3b**	460.488	4.5809	−7.615	0	6	0	4.83	76.04
**3c**	476.487	4.167	−7.289	0	7	0	4.9122	85.27
**3d**	480.906	4.843	−8.007	0	6	0	4.9183	76.04
**3e**	480.906	4.843	−8.007	0	6	0	4.9183	76.04
**3f**	515.351	5.449	−8.743	0	6	0	4.9183	76.04
**3g**	462.46	3.8913	−6.975	1	7	0	4.8783	96.27
**3h**	491.458	3.3154	−7.731	0	9	0	−0.18075	121.86
**3i**	491.458	3.3154	−7.731	0	9	0	−0.18075	121.86
**3j**	464.451	4.3378	−7.585	0	6	0	3.5334	76.04

MW < 500; LogP < 5; LogS > −4; H-donors < 5; H-acceptors < 10; Drug-likeness > 0; TPSA < 140 Å^2^.

## Data Availability

The data presented in this study are available in Supplementary Material.

## Supplementary Materials

The following supporting information can be downloaded at: https://www.mdpi.com/article/10.3390/molecules27227878/s1, Supplementary Data S1: The NMR and HRMS spectras.

## References

[1] Henidi H.A., Al-Abd A.M., Al-Abbasi F.A., BinMahfouz H.A., El-Deeb I.M. (2019). Design and Synthesis of Novel Phenylaminopyrimidines with Antiproliferative Activity against Colorectal Cancer. RSC Adv..

[2] Siegel R.L., Miller K.D., Jemal A. (2017). Cancer Statistics, 2017. CA Cancer J. Clin..

[3] Ranjan Dwivedi A., Kumar V., Kaur H., Kumar N., Prakash Yadav R., Poduri R., Baranwal S., Kumar V. (2020). Anti-Proliferative Potential of Triphenyl Substituted Pyrimidines against MDA-MB-231, HCT-116 and HT-29 Cancer Cell Lines. Bioorg. Med. Chem. Lett..

[4] Cancer. https://www.who.int/health-topics/cancer.

[5] Siripong P., Kanokmedakul K., Piyaviriyagul S., Yahuafai J., Chanpai R., Ruchirawat S., Oku N. (2006). Antiproliferative Naphthoquinone Esters from *Rhinacanthus nasutus* Kurz. Roots on Various Cancer Cells. J. Tradit. Med..

[6] Sperry J., Lorenzo-Castrillejo I., Brimble M.A., Machín F. (2009). Pyranonaphthoquinone Derivatives of Eleutherin, Ventiloquinone L, Thysanone and Nanaomycin A Possessing a Diverse Topoisomerase II Inhibition and Cytotoxicity Spectrum. Bioorg. Med. Chem..

[7] Abdelfattah M.S., Kazufumi T., Ishibashi M. (2011). New Pyranonaphthoquinones and a Phenazine Alkaloid Isolated from Streptomyces Sp. IFM 11307 with TRAIL Resistance-Overcoming Activity. J. Antibiot..

[8] Yang Z., Ding J., Ding K., Chen D., Cen S., Ge M. (2013). Phomonaphthalenone A: A Novel Dihydronaphthalenone with Anti-HIV Activity from Phomopsis Sp. HCCB04730. Phytochem. Lett..

[9] Jardosh H.H., Patel M.P. (2013). Microwave-Induced CAN Promoted Atom-Economic Synthesis of 1H-Benzo[b]Xanthene and 4H-Benzo[g]Chromene Derivatives of N-Allyl Quinolone and Their Antimicrobial Activity. Med. Chem. Res..

[10] Tangeti V., Vasundhara D., Kumar M., Mylapalli H., Kumar K. (2017). Synthesis, Characterization and Cytotoxic Investigations of Novel C3-Dihydrofuran Substituted 1H-Benzo[g]Chromene-2,5,10-Triones besides Antimicrobial Study. Asian J. Chem..

[11] Guimarães T.T., Pinto M.d.C.F.R., Lanza J.S., Melo M.N., do Monte-Neto R.L., de Melo I.M.M., Diogo E.B.T., Ferreira V.F., Camara C.A., Valença W.O. (2013). Potent Naphthoquinones against Antimony-Sensitive and -Resistant Leishmania Parasites: Synthesis of Novel α- and nor-α-Lapachone-Based 1,2,3-Triazoles by Copper-Catalyzed Azide–Alkyne Cycloaddition. Eur. J. Med. Chem..

[12] Al Nasr I.S., Jentzsch J., Shaikh A., Singh Shuveksh P., Koko W.S., Khan T.A., Ahmed K., Schobert R., Ersfeld K., Biersack B. (2021). New Pyrano-4H-Benzo[g]Chromene-5,10-Diones with Antiparasitic and Antioxidant Activities. Chem. Biodivers..

[13] Kumar S., Malachowski W.P., DuHadaway J.B., LaLonde J.M., Carroll P.J., Jaller D., Metz R., Prendergast G.C., Muller A.J. (2008). Indoleamine 2,3-Dioxygenase Is the Anticancer Target for a Novel Series of Potent Naphthoquinone-Based Inhibitors. J. Med. Chem..

[14] da Rocha D.R., de Souza A.C.G., Resende J.A.L.C., Santos W.C., dos Santos E.A., Pessoa C., de Moraes M.O., Costa-Lotufo L.V., Montenegro R.C., Ferreira V.F. (2011). Synthesis of New 9-Hydroxy-α- and 7-Hydroxy-β-Pyran Naphthoquinones and Cytotoxicity against Cancer Cell Lines. Org. Biomol. Chem..

[15] Magedov I.V., Kireev A.S., Jenkins A.R., Evdokimov N.M., Lima D.T., Tongwa P., Altig J., Steelant W.F.A., Van Slambrouck S., Antipin M.Y. (2012). Structural Simplification of Bioactive Natural Products with Multicomponent Synthesis. 4. 4H-Pyrano-[2,3-b]Naphthoquinones with Anticancer Activity. Bioorg. Med. Chem. Lett..

[16] da Cruz E.H.G., Silvers M.A., Jardim G.A.M., Resende J.M., Cavalcanti B.C., Bomfim I.S., Pessoa C., de Simone C.A., Botteselle G.V., Braga A.L. (2016). Synthesis and Antitumor Activity of Selenium-Containing Quinone-Based Triazoles Possessing Two Redox Centres, and Their Mechanistic Insights. Eur. J. Med. Chem..

[17] Ravichandiran P., Subramaniyan S.A., Kim S.-Y., Kim J.-S., Park B.-H., Shim K.S., Yoo D.J. (2019). Synthesis and Anticancer Evaluation of 1,4-Naphthoquinone Derivatives Containing a Phenylaminosulfanyl Moiety. ChemMedChem.

[18] Patel R.B., Desai P.S., Desai K.R., Chikhalia K.H. (2006). Synthesis of Pyrimidine Based Thiazolidinones and Azetidinones: Antimicrobial and Antitubercular Agents. IJC-B.

[19] Rostom S.A.F., Ashour H.M.A., Abd El Razik H.A. (2009). Synthesis and Biological Evaluation of Some Novel Polysubstituted Pyrimidine Derivatives as Potential Antimicrobial and Anticancer Agents. Archiv. Pharm..

[20] Garavito M.F., Narváez-Ortiz H.Y., Zimmermann B.H. (2015). Pyrimidine Metabolism: Dynamic and Versatile Pathways in Pathogens and Cellular Development. J. Genet. Genom..

[21] Stolarczyk M., Wolska A., Mikołajczyk A., Bryndal I., Cieplik J., Lis T., Matera-Witkiewicz A. (2021). A New Pyrimidine Schiff Base with Selective Activities against Enterococcus Faecalis and Gastric Adenocarcinoma. Molecules.

[22] Sondhi S.M., Singh N., Johar M., Kumar A. (2005). Synthesis, Anti-Inflammatory and Analgesic Activities Evaluation of Some Mono, Bi and Tricyclic Pyrimidine Derivatives. Bioorg. Med. Chem..

[23] Abu-Hashem A.A., Gouda M.A., Badria F.A. (2010). Synthesis of Some New Pyrimido[2′,1′:2,3]Thiazolo[4,5-b]Quinoxaline Derivatives as Anti-Inflammatory and Analgesic Agents. Eur. J. Med. Chem..

[24] Abdellatif K.R.A., Abdelall E.K.A., Abdelgawad M.A., Ahmed R.R., Bakr R.B. (2014). Synthesis and Anticancer Activity of Some New Pyrazolo[3,4-d]Pyrimidin-4-One Derivatives. Molecules.

[25] Shyyka O., Pokhodylo N., Finiuk N., Matiychuk V., Stoika R., Obushak M. (2018). Anticancer Activity Evaluation of New Thieno[2,3-d]Pyrimidin-4(3H)-Ones and Thieno[3,2-d]Pyrimidin-4(3H)-One Derivatives. Sci. Pharm..

[26] Fouad M.M., El-Bendary E.R., Suddek G.M., Shehata I.A., El-Kerdawy M.M. (2018). Synthesis and in Vitro Antitumor Evaluation of Some New Thiophenes and Thieno[2,3-d]Pyrimidine Derivatives. Bioorg. Chem..

[27] Cherukupalli S., Chandrasekaran B., Aleti R.R., Sayyad N., Hampannavar G.A., Merugu S.R., Rachamalla H.R., Banerjee R., Karpoormath R. (2019). Synthesis of 4,6-Disubstituted Pyrazolo[3,4-d]Pyrimidine Analogues: Cyclin-Dependent Kinase 2 (CDK2) Inhibition, Molecular Docking and Anticancer Evaluation. J. Mol. Struct..

[28] Kilic A., Beyazsakal L., Işık M., Türkeş C., Necip A., Takım K., Beydemir Ş. (2020). Mannich Reaction Derived Novel Boron Complexes with Amine-Bis(Phenolate) Ligands: Synthesis, Spectroscopy and in Vitro/in Silico Biological Studies. J. Organomet. Chem..

[29] Malki A., Ashour H.M.A., Elbayaa R.Y., Issa D.A.E., Aziz H.A., Chen X. (2016). Novel 1,5-Diphenyl-6-Substituted 1H-Pyrazolo[3,4-d]Pyrimidin-4(5H)-Ones Induced Apoptosis in RKO Colon Cancer Cells. J. Enzym. Inhib. Med. Chem..

[30] Dgachi Y., Martin H., Bonet A., Chioua M., Iriepa I., Moraleda I., Chabchoub F., Marco-Contelles J., Ismaili L. (2017). Synthesis and Biological Assessment of Racemic Benzochromenopyrimidinetriones as Promising Agents for Alzheimer’s Disease Therapy. Future Med. Chem..

[31] Cherif M., Horchani M., Al-Ghamdi Y.O., Almalki S.G., Alqurashi Y.E., Ben Jannet H., Romdhane A. (2020). New Pyrano-1,2,3-Triazolopyrimidinone Derivatives as Anticholinesterase and Antibacterial Agents: Design, Microwave-Assisted Synthesis and Molecular Docking Study. J. Mol. Struct..

[32] Khurana J.M., Lumb A., Chaudhary A., Nand B. (2013). Synthesis and in Vitro Evaluation of Antioxidant Activity of Diverse Naphthopyranopyrimidines, Diazaanthra[2,3-d][1,3]Dioxole-7,9-Dione and Tetrahydrobenzo[a]Xanthen-11-Ones. RSC Adv..

[33] Mobinikhaledi A., Foroughifar N., Mosleh T., Hamta A. (2014). Synthesis of Some Novel Chromenopyrimidine Derivatives and Evaluation of Their Biological Activities. Iran J. Pharm. Res..

[34] Ravichandiran P., Sheet S., Premnath D., Kim A.R., Yoo D.J. (2019). 1,4-Naphthoquinone Analogues: Potent Antibacterial Agents and Mode of Action Evaluation. Molecules.

[35] Ravichandiran P., Masłyk M., Sheet S., Janeczko M., Premnath D., Kim A.R., Park B.-H., Han M.-K., Yoo D.J. (2019). Synthesis and Antimicrobial Evaluation of 1,4-Naphthoquinone Derivatives as Potential Antibacterial Agents. Chem. Open.

[36] Khurana J.M., Nand B., Saluja P. (2010). DBU: A Highly Efficient Catalyst for One-Pot Synthesis of Substituted 3,4-Dihydropyrano[3,2-c]Chromenes, Dihydropyrano[4,3-b]Pyranes, 2-Amino-4H-Benzo[h]Chromenes and 2-Amino-4H Benzo[g]Chromenes in Aqueous Medium. Tetrahedron.

[37] Yu Y., Guo H., Li X. (2011). An Improved Procedure for the Three-Component Synthesis of Benzo[g]Chromene Derivatives Using Basic Ionic Liquid. J. Heterocycl. Chem..

[38] Khurana J.M., Magoo D., Chaudhary A. (2012). Efficient and Green Approaches for the Synthesis of 4H-Benzo[g]Chromenes in Water, Under Neat Conditions, and Using Task-Specific Ionic Liquid. Synth. Commun..

[39] Dekamin M.G., Eslami M., Maleki A. (2013). Potassium Phthalimide-N-Oxyl: A Novel, Efficient, and Simple Organocatalyst for the One-Pot Three-Component Synthesis of Various 2-Amino-4H-Chromene Derivatives in Water. Tetrahedron.

[40] Rahimzadeh G., Tajbakhsh M., Daraie M., Mohammadi M. (2022). Dysprosium–Balsalazide Complex Trapped between the Functionalized Halloysite and g-C_3_N_4_: A Novel Heterogeneous Catalyst for the Synthesis of Annulated Chromenes in Water. Appl. Organomet. Chem..

[41] Safaei-Ghomi J., Bateni F.-S., Babaei P. (2020). CeO_2_/CuO@N-GQDs@NH_2_ Nanocomposite as a High-Performance Catalyst for the Synthesis of Benzo[g]Chromenes. Appl. Organomet. Chem..

[42] Safaei-Ghomi J., Enayat-Mehri N., Eshteghal F. (2018). 4-(4′-Diamino-Di-Phenyl)-Sulfone Supported on Hollow Magnetic Mesoporous Fe_3_O_4_@SiO_2_ NPs: As a Reusable and Efficient Catalyst for the Synthesis of Ethyl 2-Amino-5,10-Dihydro-5,10-Dioxo-4-Phenyl-4H Benzo[g]Chromene-3-Carboxylates. J. Saudi Chem. Soc..

