# Supplementary material for: Benzochromenopyrimidines: Synthesis, Antiproliferative Activity against Colorectal Cancer and Physicochemical Properties
Source: Molecules. 2022 Nov 15;27(22):7878. doi: 10.3390/molecules27227878 (PMC9694646; doi:10.3390/molecules27227878)
Supplement: Supplementary file 1 [file molecules-27-07878-s001.zip › molecules-1973707-supplementary.pdf]

SUPPLEMENTARY DATA

NMR and HRMS spectra of compounds **3a-j**

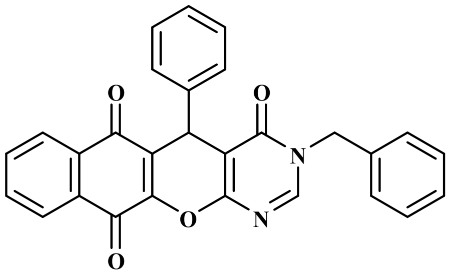

3-Benzyl-5-phenyl-3,5-dihydro-4*H*-benzo[6,7]chromeno[2,3-*d*]pyrimidine-4,6,11-trione (3a): It was obtained as a light yellow solid, yield: 59%, m.p.: > 260 °C. ^{1}H NMR (400 MHz, DMSO-*d6*) δ_H (ppm): 8.73 (s, 1H, C$\underline{H}_{pyrimidine}$), 8.10-8.07 (m, 1H), 7.93-7.91 (m, 1H), 7.89-7.85 (m, 2H), 7.38 (d, *J*= 7.4 Hz, 2H), 7.32-7.24 (m, 7H), 7.19-7.15 (m, 1H), 5.14 (d, *J*= 14.7 Hz, 1H, CH_2), 5.13 (s, 1H, C$\underline{H}_{pyran}$), 4.98 (d, *J*= 14.7 Hz, 1H, CH_2). ^{13}C NMR (100 MHz, DMSO-*d6*) δ_C (ppm): 183.0, 177.5, 160.4, 159.7, 152.2, 150.1, 142.4, 136.4, 135.1, 134.7, 131.5, 131.1, 129.1 (2C), 129.0 (2C), 128.7 (2C), 128.3, 128.2 (2C), 127.5, 126.6, 126.3, 123.3, 103.7, 49.8, 34.8. HRMS (ESI, M+H$^+$): calcd for $C_{28}H_{19}N_2O_4$; 447.1345, found; 447.1339.

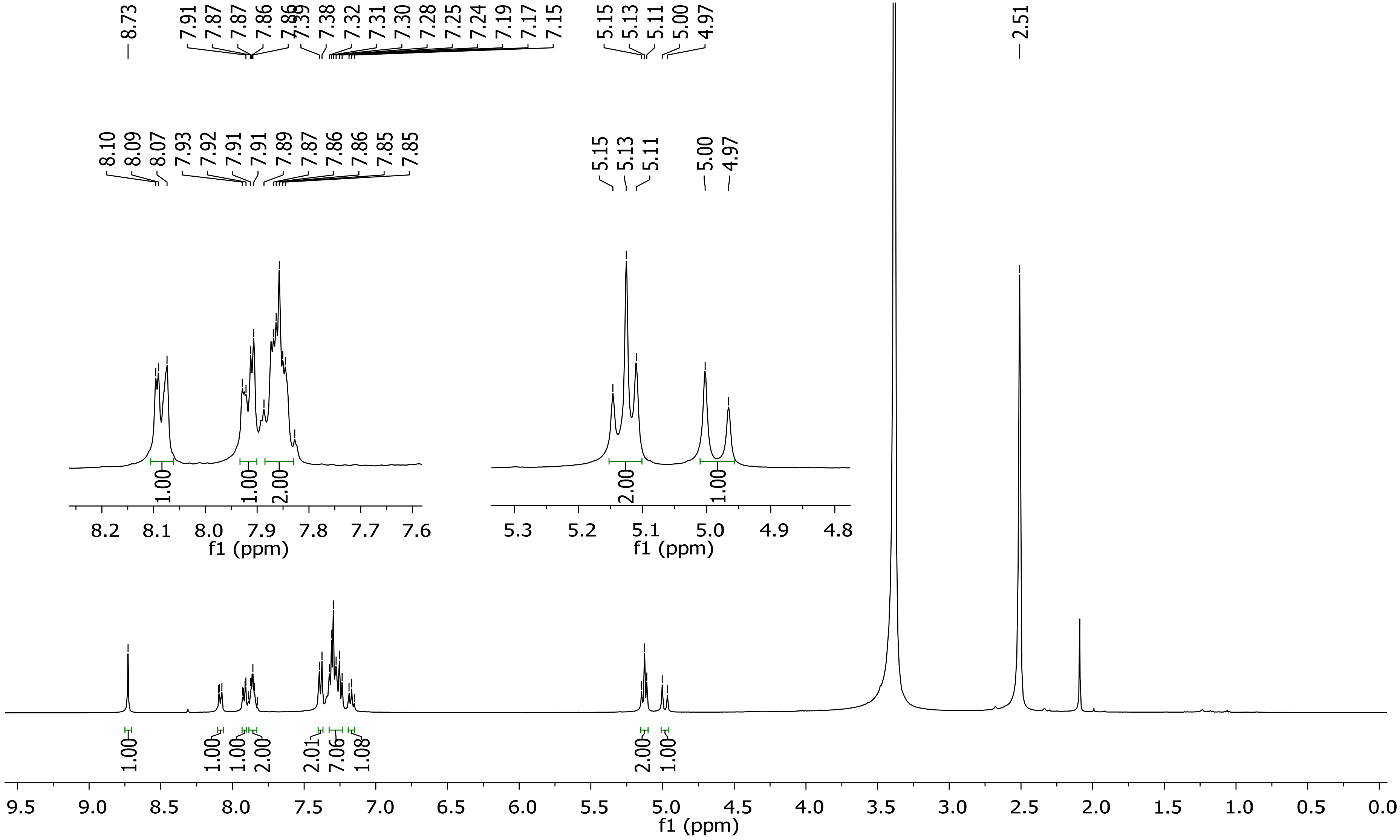

^{1}H NMR of compound **3a** (400 MHz, DMSO-d_6)

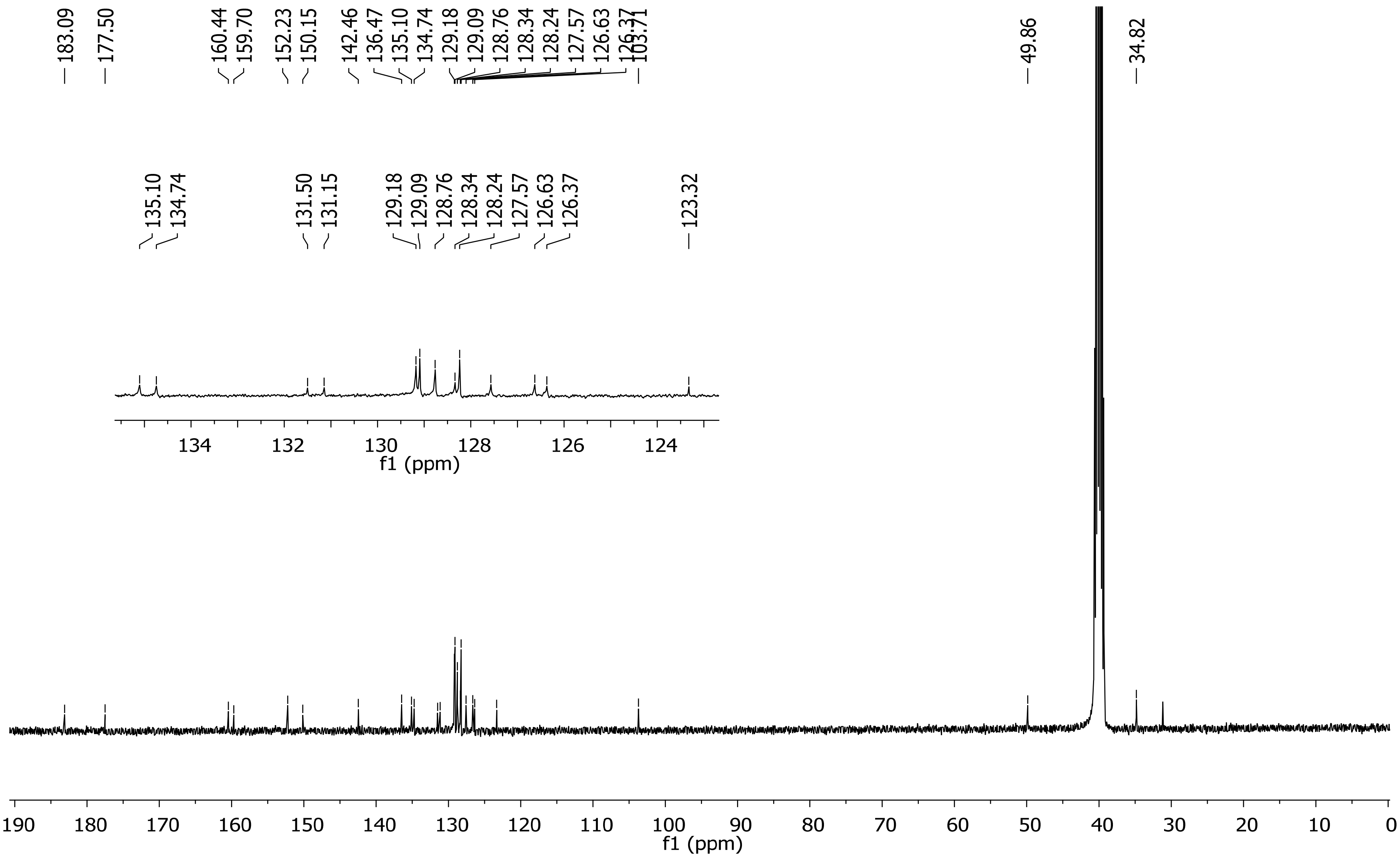

^{13}C NMR of compound **3a** (100 MHz, DMSO-d_6)

D:\Data\2022\LINC\EmCh-8-A.raw 6/9/2022 8:29:42 AM

EmCh-8-A #81 RT: 0.79 AV: 1 NL: 1.12E8
T: FTMS + p ESI Full ms [50.0000-750.0000]

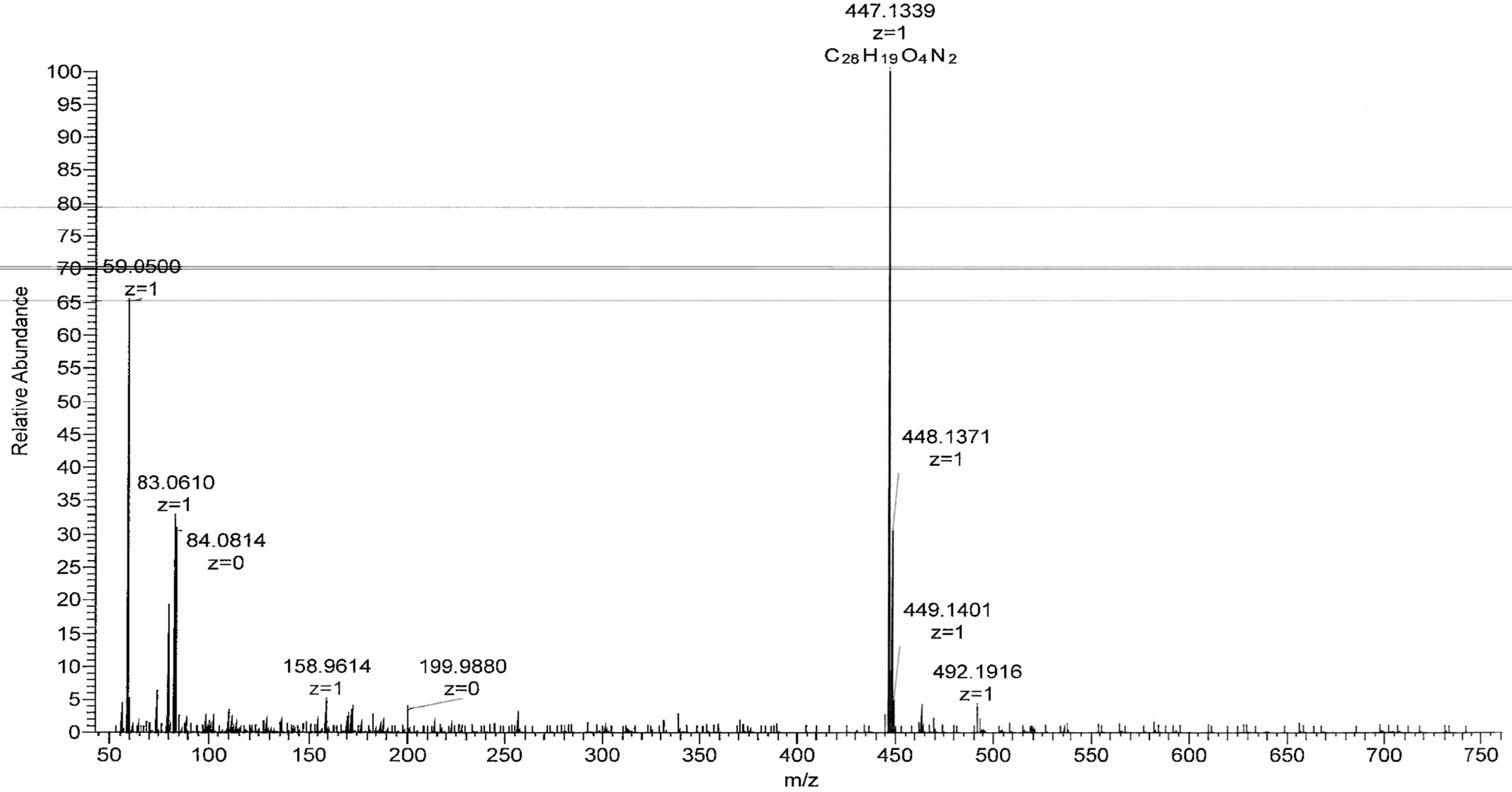

Peak Mass	Display Formula	Delta [ppm]	Theo. mass	Combined Score	Pattern Cov. [%]	MSMS Matched Fragments
447.1339	$C_{28}H_{19}O_4N_2$	-0.19	447.13393	99.12	100	(Collection)

HRMS spectrum of compound **3a** (ESI, M+H$^+$)

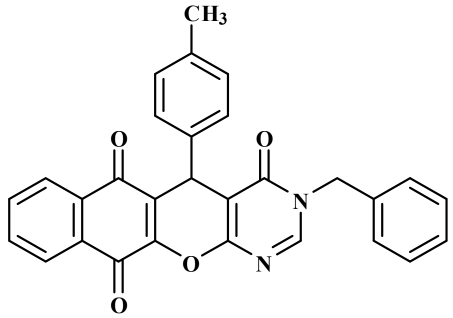

3-Benzyl-5-(*p*-tolyl)-3,5-dihydro-4*H*-benzo[6,7]chromeno[2,3-*d*]pyrimidine-4,6,11-trione (3b): It was obtained as an olive green solid, yield: 53%, m.p.: 252-254 °C. ^{1}H NMR (400 MHz, DMSO-*d6*) δ_H (ppm): 8.71 (s, 1H, C$\underline{H}_{pyrimidine}$), 8.09-8.07 (m, 1H), 7.90-7.86 (m, 3H), 7.30-7.24 (m, 7H), 7.05 (d, *J*= 7.1 Hz, 2H), 5.12 (d, *J*= 14.7 Hz, 1H, CH_2), 5.08 (s, 1H, C$\underline{H}_{pyran}$), 4.98 (d, *J*= 14.7 Hz, 1H, CH_2), 2.20 (s, 3H, CH_3). ^{13}C NMR (100 MHz, DMSO-*d6*) δ_C (ppm): 183.0, 177.5, 160.4, 159.6, 152.1, 149.9, 139.5, 136.8, 136.4, 135.1, 134.7, 131.5, 131.0, 129.3 (2C), 129.1 (2C), 129.0 (2C), 128.3, 128.2 (2C), 126.6, 126.3, 123.5, 100.8, 49.8, 34.3, 21.0. HRMS (ESI, M+H$^+$): calcd for $C_{29}H_{21}N_2O_4$; 461.1501, found; 461.1494.

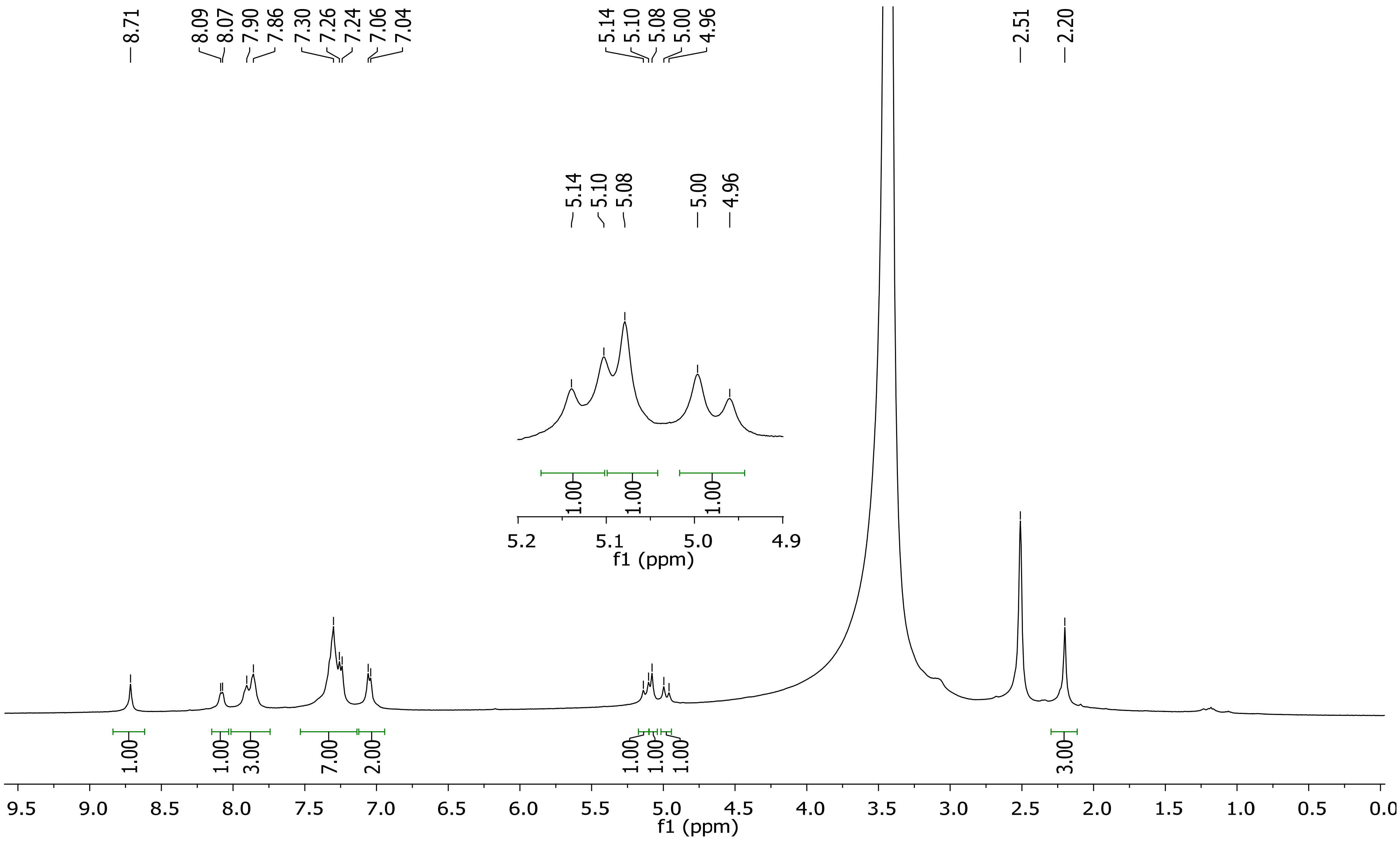

^{1}H NMR of compound **3b** (400 MHz, DMSO-d_6)

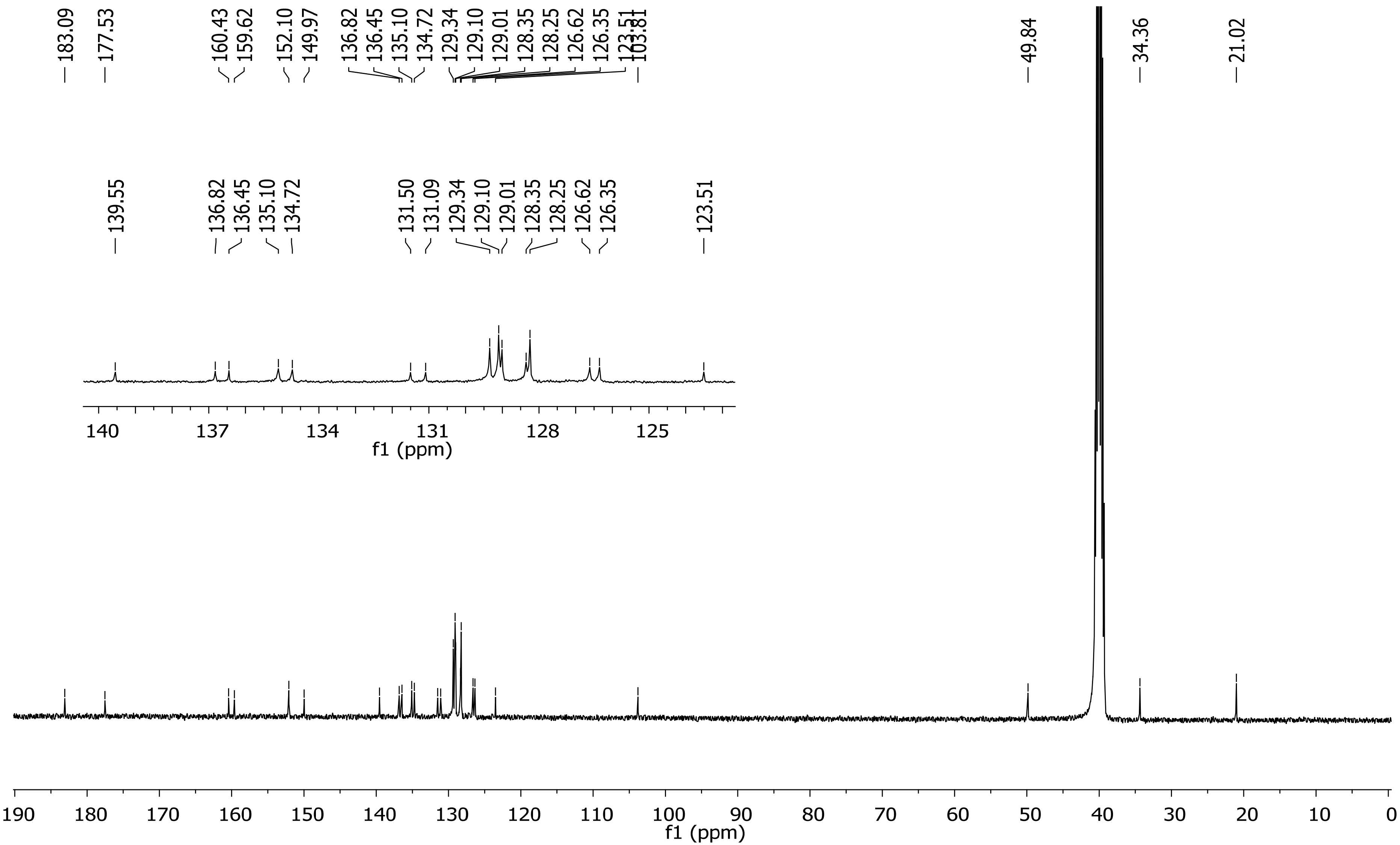

^{13}C NMR of compound **3b** (100 MHz, DMSO-d_6)

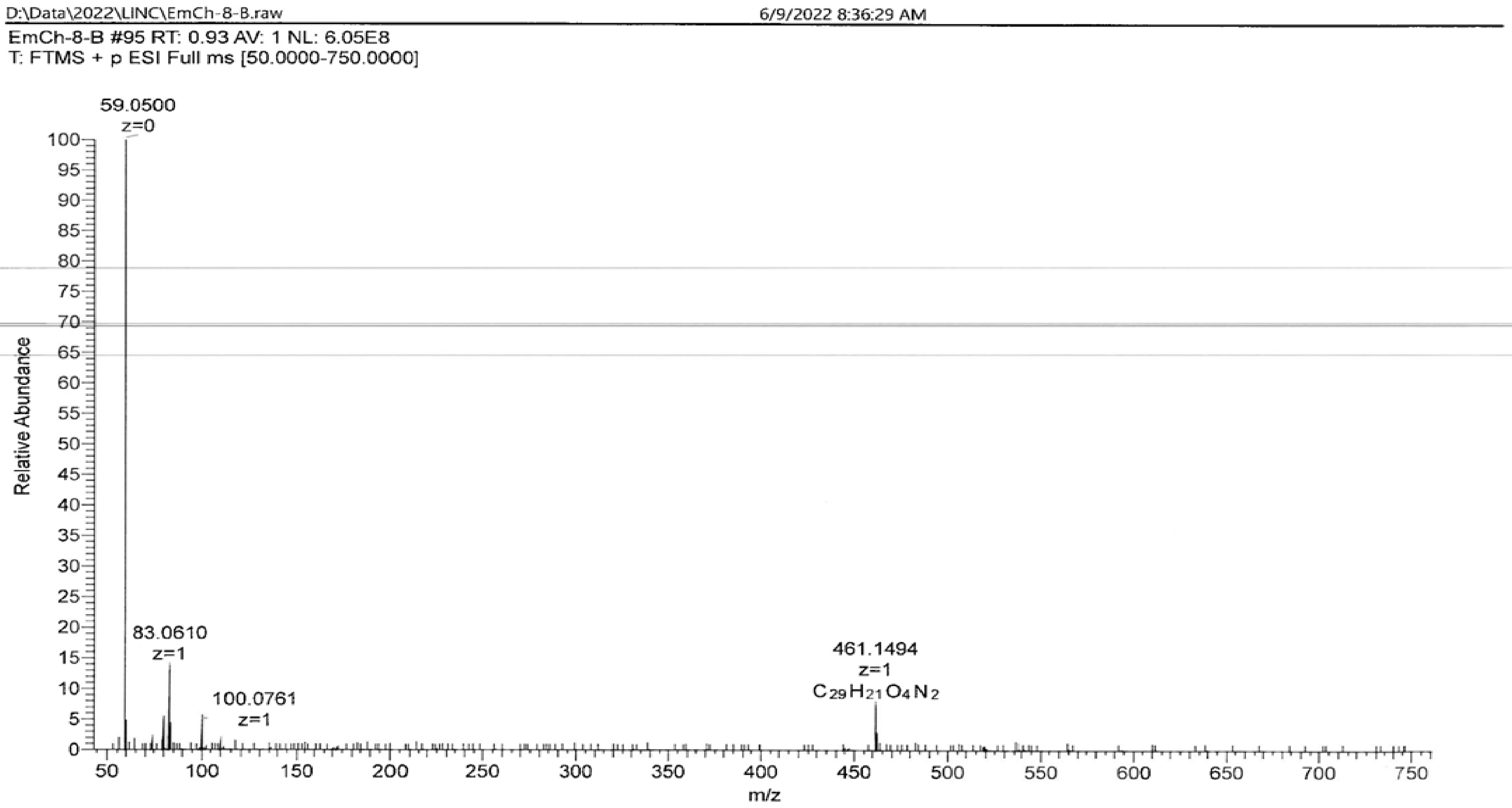

Peak Mass	Display Formula	Delta [ppm]	Theo. mass	Combined Score	Pattern Cov. [%]	MSMS Matched Fragments
461.1494	$C_{29}H_{21}O_4N_2$	-0.43	461.14958	96.44	99.49	(Collection)

HRMS spectrum of compound **3b** (ESI, M+H$^+$)

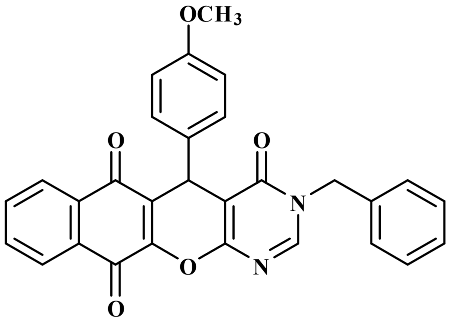

3-Benzyl-5-(4-methoxyphenyl)-3,5-dihydro-4*H*-benzo[6,7]chromeno[2,3-*d*]pyrimidine-4,6,11-trione (3c): It was obtained as an ochre yellow solid, yield: 60%, m.p.: > 260 °C. ^{1}H NMR (400 MHz, DMSO-*d6*) δ_H (ppm): 8.71 (s, 1H, C$\underline{H}_{pyrimidine}$), 8.09-8.07 (m, 1H), 7.92-7.86 (m, 3H), 7.31 (m, 7H), 6.80 (d, *J*= 7.6 Hz, 2H), 5.12 (d, *J*= 14.2 Hz, 1H, CH_2), 5.07 (s, 1H, C$\underline{H}_{pyran}$), 4.98 (d, *J*= 14.2 Hz, 1H, CH_2), 3.68 (s, 3H, OCH_3). ^{13}C NMR (100 MHz, DMSO-*d6*) δ_C (ppm): 183.1, 177.5, 160.4, 159.5, 158.7, 152.0, 149.8, 136.4, 135.1, 134.7, 134.6, 131.5, 131.1, 130.2 (2C), 129.1 (2C), 128.3, 128.2 (2C), 126.6, 126.3, 123.4, 114.1 (2C), 103.8, 55.4, 49.8, 33.9. HRMS (ESI, M+H$^+$): calcd for $C_{29}H_{21}N_2O_5$; 477.1450, found; 477.1443.

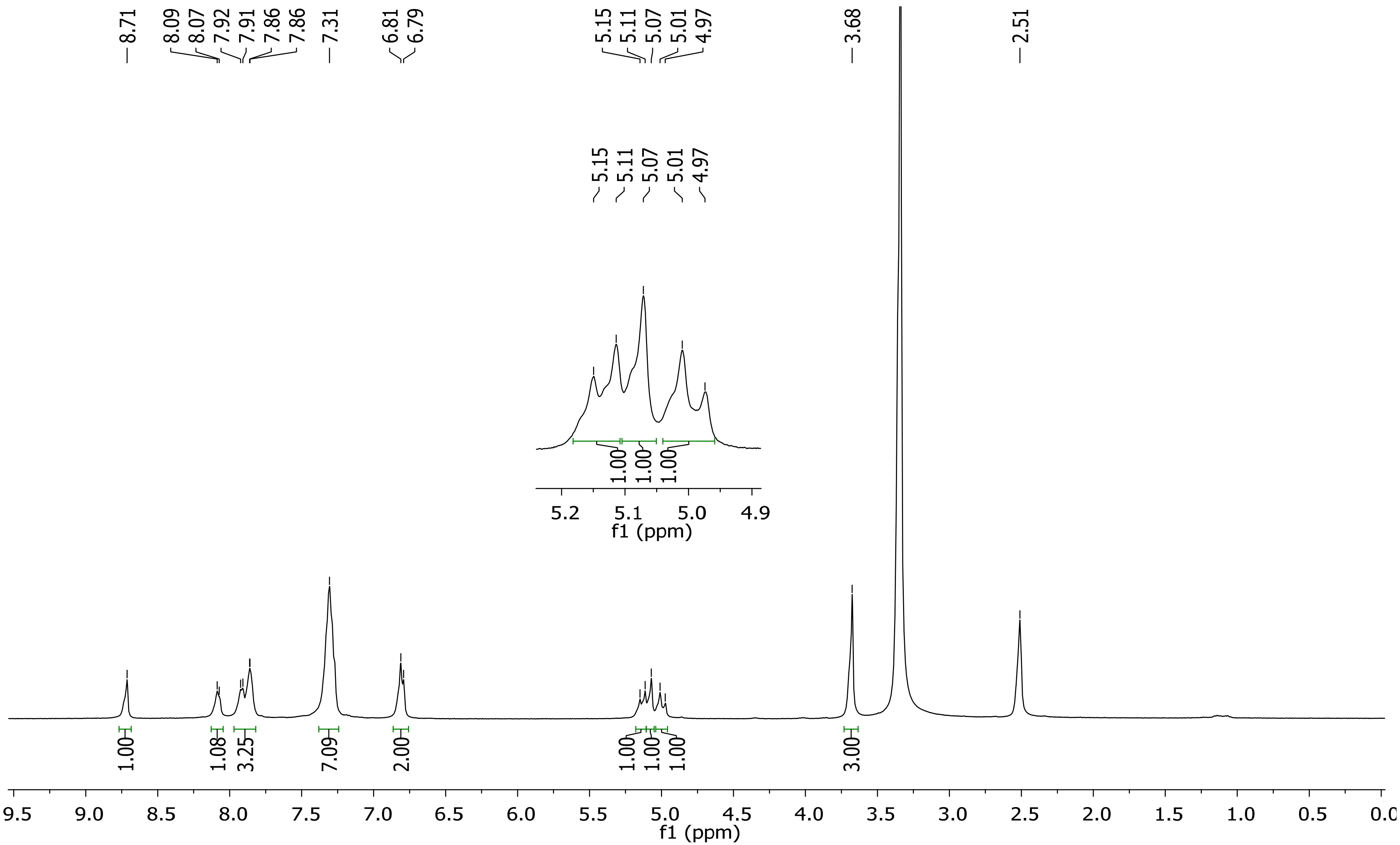

^{1}H NMR of compound **3c** (400 MHz, DMSO-*d*$_6$)

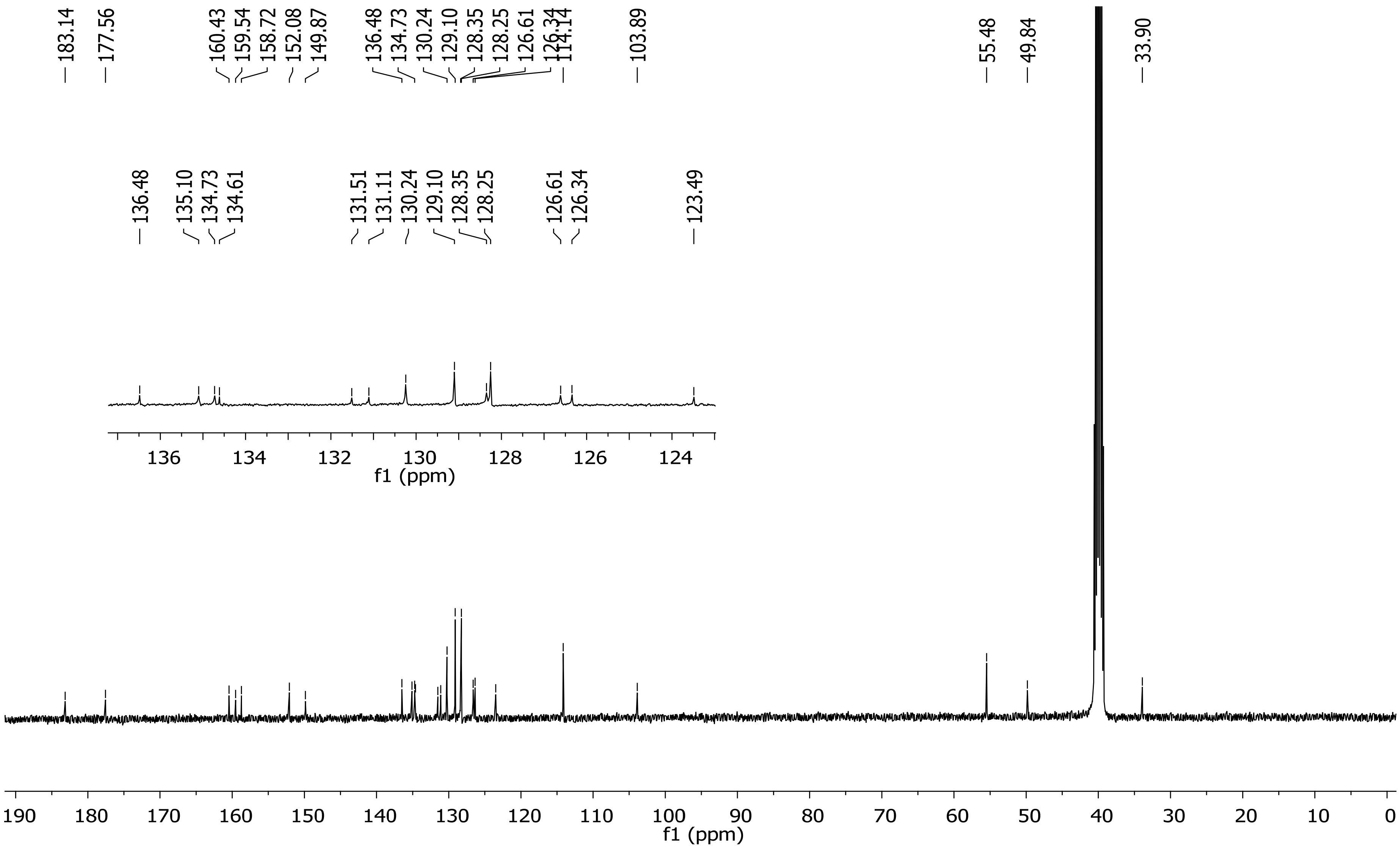

^{13}C NMR of compound **3c** (100 MHz, DMSO-d_6)

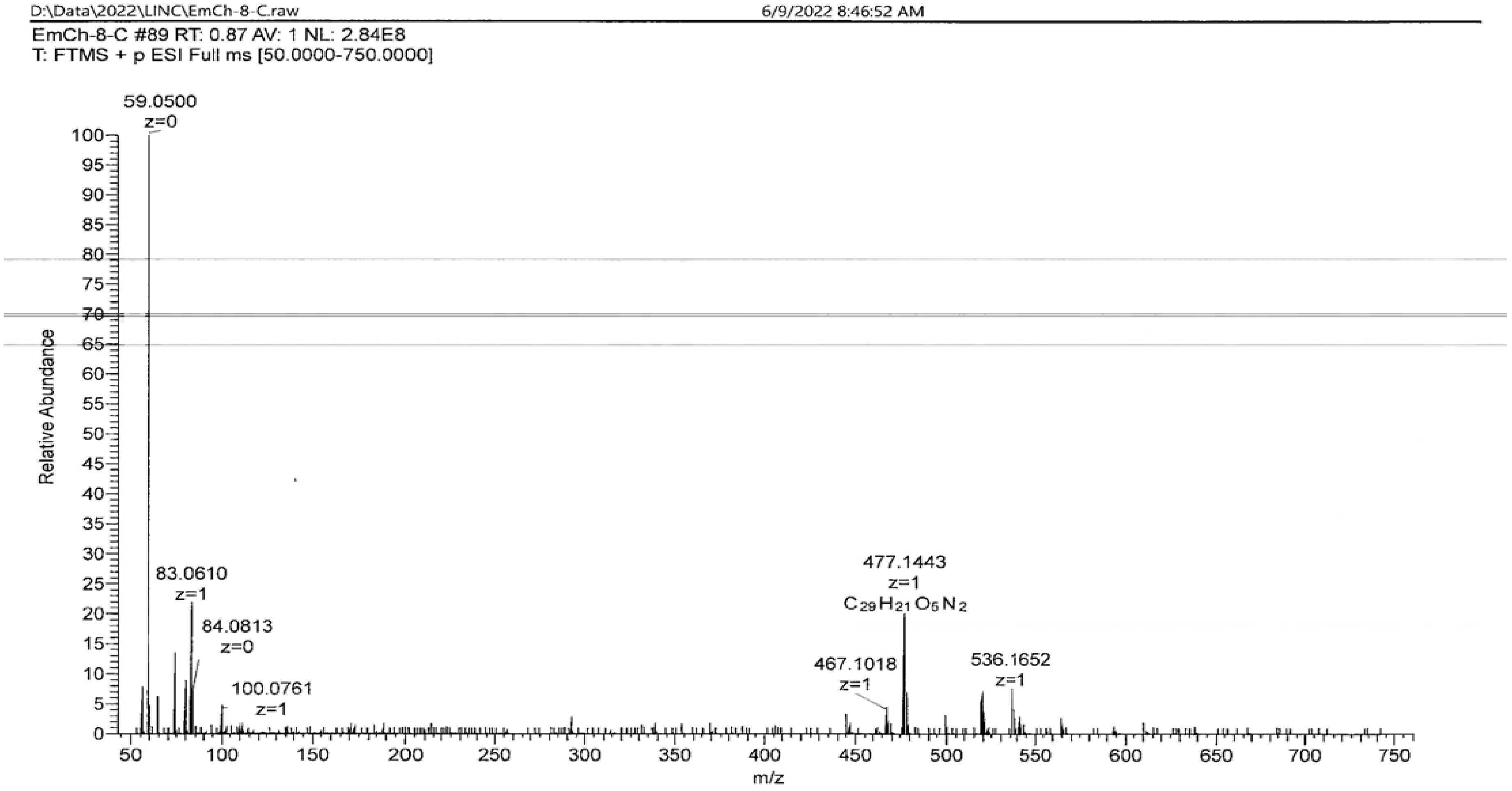

Peak Mass	Display Formula	Delta [ppm]	Theo. mass	Combined Score	Pattern Cov. [%]	MSMS Matched Fragments
477.1443	$C_{29}H_{21}O_5N_2$	-0.38	477.14450	98.57	100	(Collection)

HRMS spectrum of compound **3c** (ESI, $M+H^+$)

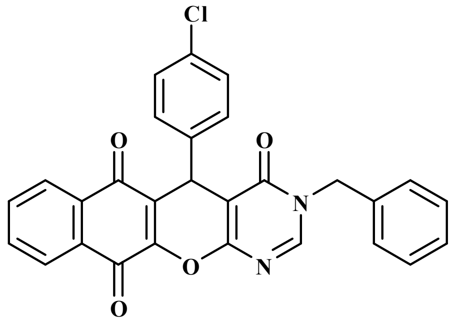

3-Benzyl-5-(4-chlorophenyl)-3,5-dihydro-4*H*-benzo[6,7]chromeno[2,3-*d*]pyrimidine-4,6,11-trione (3d): It was obtained as a pale yellow solid, yield: 55%, m.p.: > 260 °C. ^{1}H NMR (400 MHz, DMSO-*d6*) δ_H (ppm): 8.73 (s, 1H, C$\underline{H}_{pyrimidine}$), 8.08 (m, 1H), 7.90-7.86 (m, 3H), 7.42 (d, *J*= 6.7 Hz, 2H), 7.31 (m, 7H), 5.14-5.11 (m, 2H, CH_2, C$\underline{H}_{pyran}$), 4.98 (d, *J*= 14.6 Hz, 1H, CH_2). ^{13}C NMR (100 MHz, DMSO-*d6*) δ_C (ppm): 183.0, 177.4, 160.4, 159.6, 152.3, 150.2, 141.4, 136.4, 135.0, 134.7, 132.1 (2C), 131.4, 131.1 (2C), 129.0 (2C), 128.6 (2C), 128.3, 128.2 (2C), 126.6, 126.3, 122.6, 103.2, 49.8, 34.5. HRMS (ESI, M+H$^+$): calcd for $C_{28}H_{18}{}^{35}ClN_2O_4$; 481.0955, found; 481.0948.

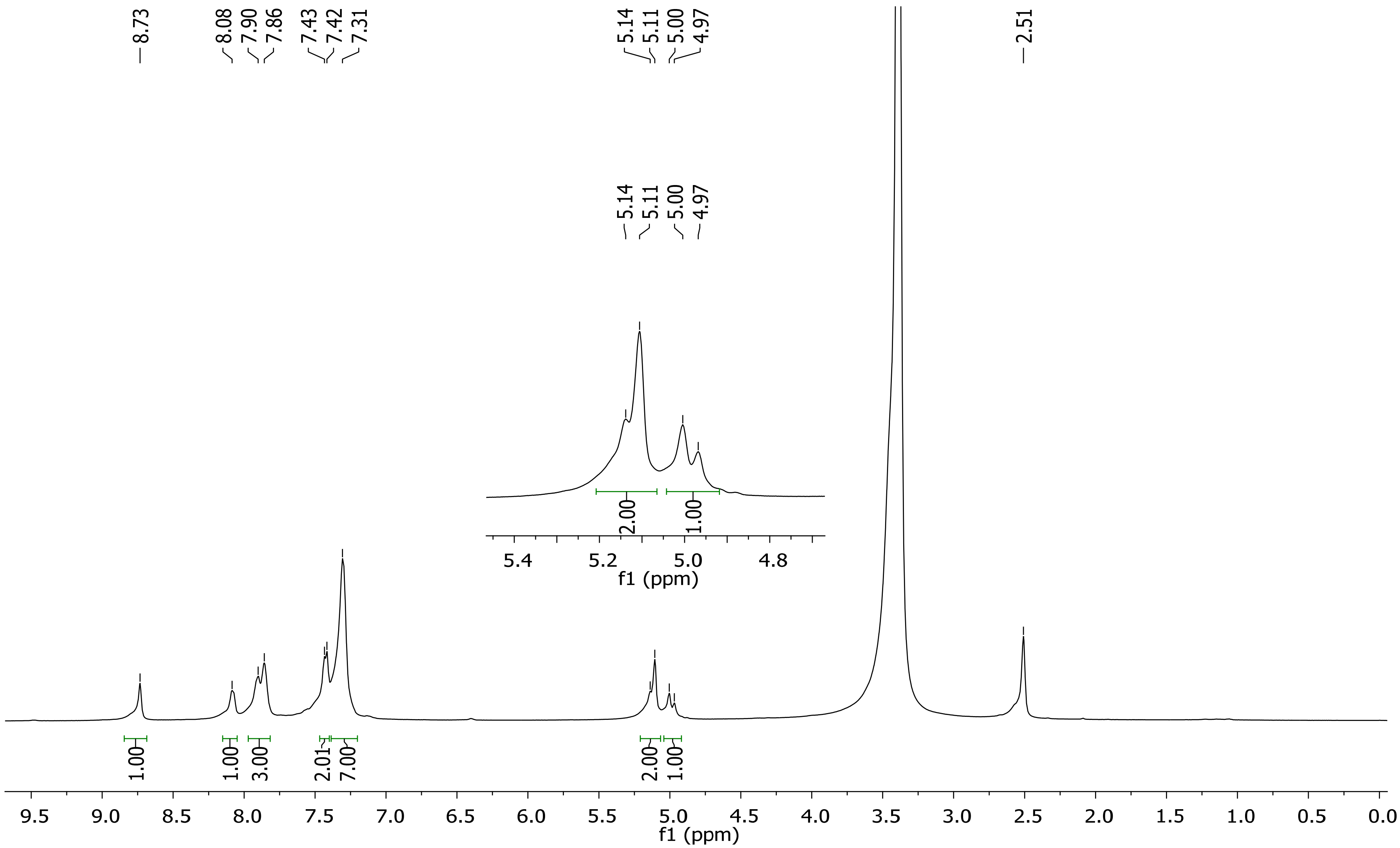

^{1}H NMR of compound **3d** (400 MHz, DMSO-*d*$_6$)

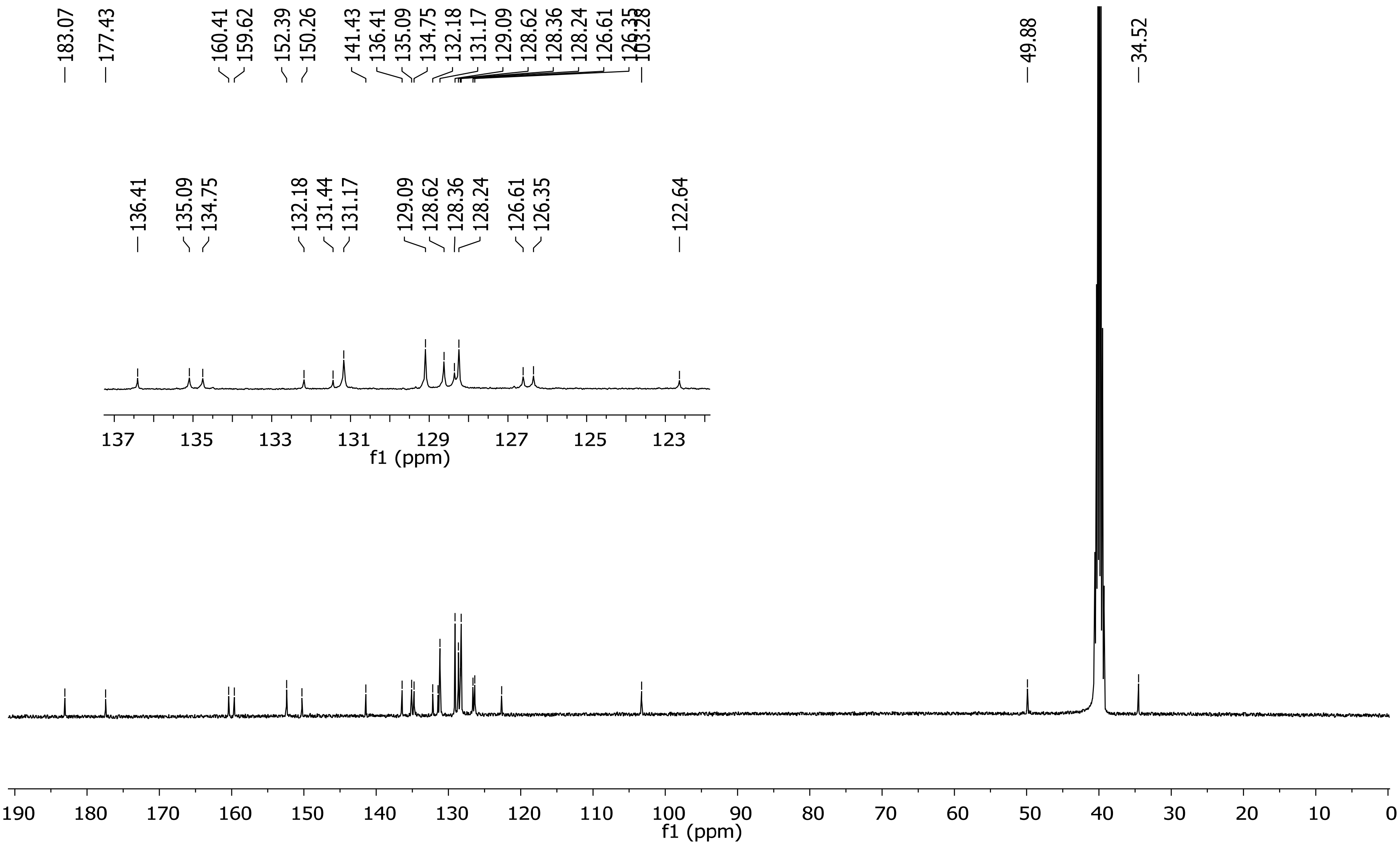

^{13}C NMR of compound **3d** (100 MHz, DMSO-d_6)

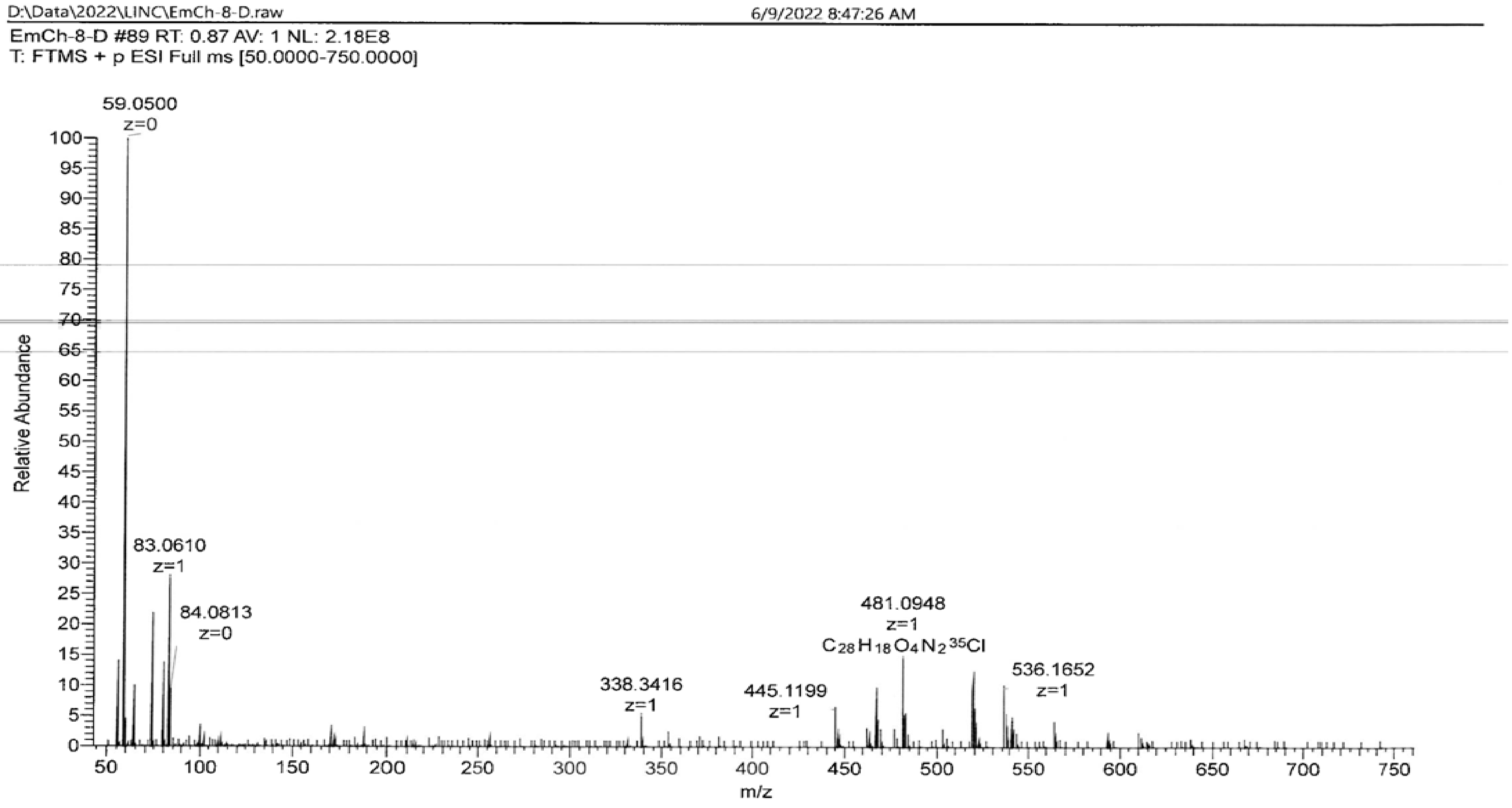

Peak Mass	Display Formula	Delta [ppm]	Theo. mass	Combined Score	Pattern Cov. [%]	MSMS Matched Fragments
481.0948	$C_{28}H_{18}O_4N_2{}^{35}Cl$	-0.23	481.09496	96.88	99.88	(Collection)

HRMS spectrum of compound **3d** (ESI, M+H$^+$)

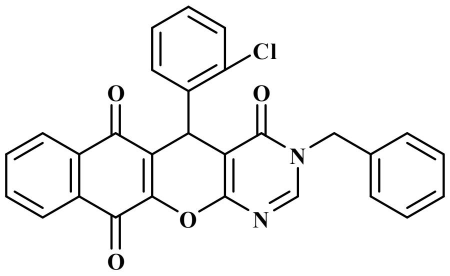

3-Benzyl-5-(2-chlorophenyl)-3,5-dihydro-4*H*-benzo[6,7]chromeno[2,3-*d*]pyrimidine-4,6,11-trione (3e): It was obtained as a red solid brick, yield: 43%, m.p.: > 260 °C. ^{1}H NMR (400 MHz, DMSO-*d6*) δ_H (ppm): 8.73 (s, 1H, C$\underline{H}_{pyrimidine}$), 8.08-8.06 (m, 1H), 7.88-7.84 (m, 3H), 7.43-7.41 (m, 2H), 7.34-7.18 (m, 7H), 5.48 (s, 1H, C$\underline{H}_{pyran}$), 5.10 (d, *J*= 14.6 Hz, 1H, CH_2), 4.95 (d, *J*= 14.6 Hz, 1H, CH_2). ^{13}C NMR (100 MHz, DMSO-*d6*) δ_C (ppm): 182.9, 177.5, 160.1, 159.8, 152.4, 150.2, 139.8, 136.4, 135.2, 134.7, 133.9, 132.7, 131.4, 130.8, 129.8 (2C), 129.1 (2C), 128.3, 128.0 (2C), 127.6, 126.5, 126.4, 122.5, 102.9, 49.7, 33.5. HRMS (ESI, M+H$^+$): calcd for $C_{28}H_{18}{}^{35}ClN_2O_4$; 481.0955, found; 481.0948.

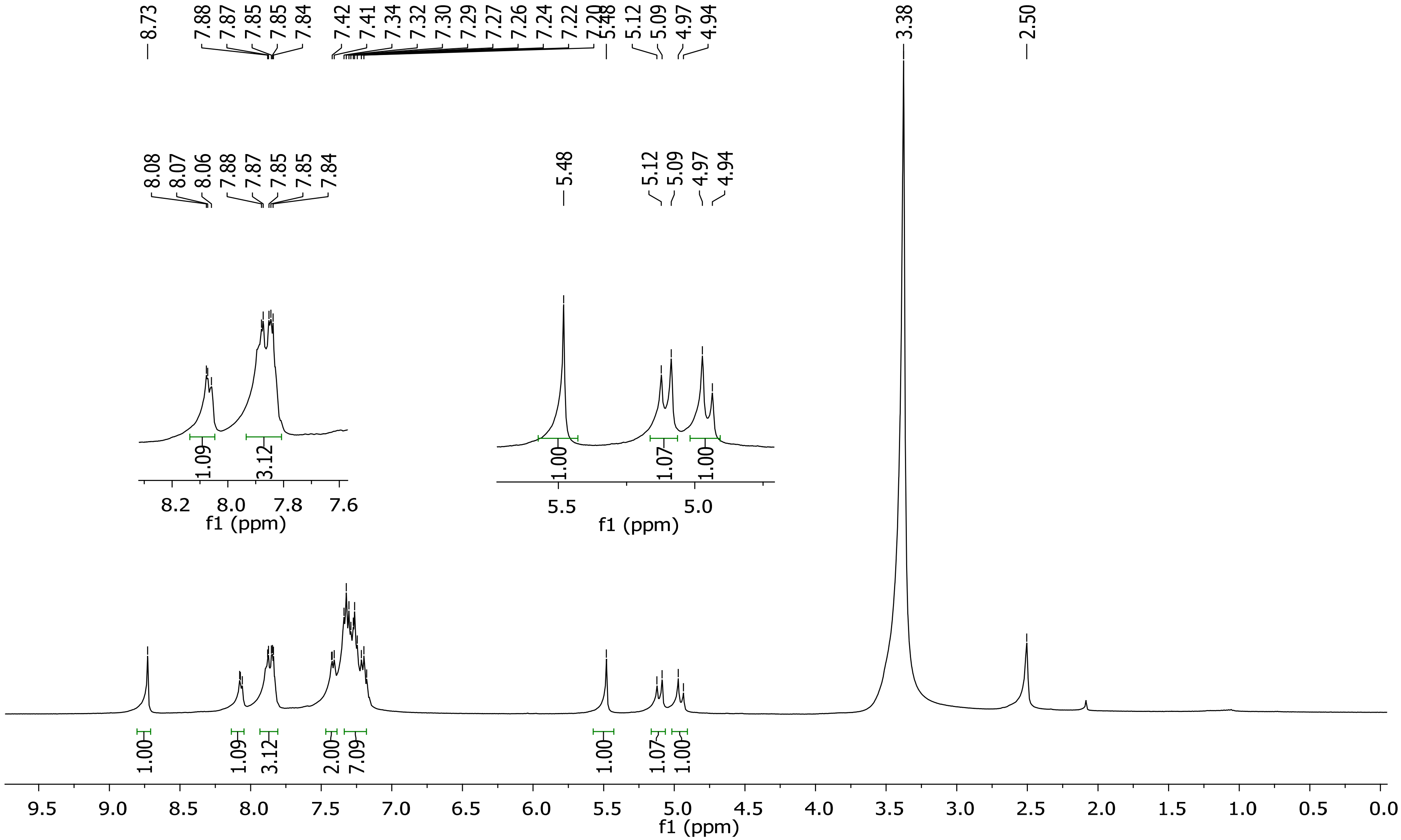

^{1}H NMR of compound **3e** (400 MHz, DMSO-d_6)

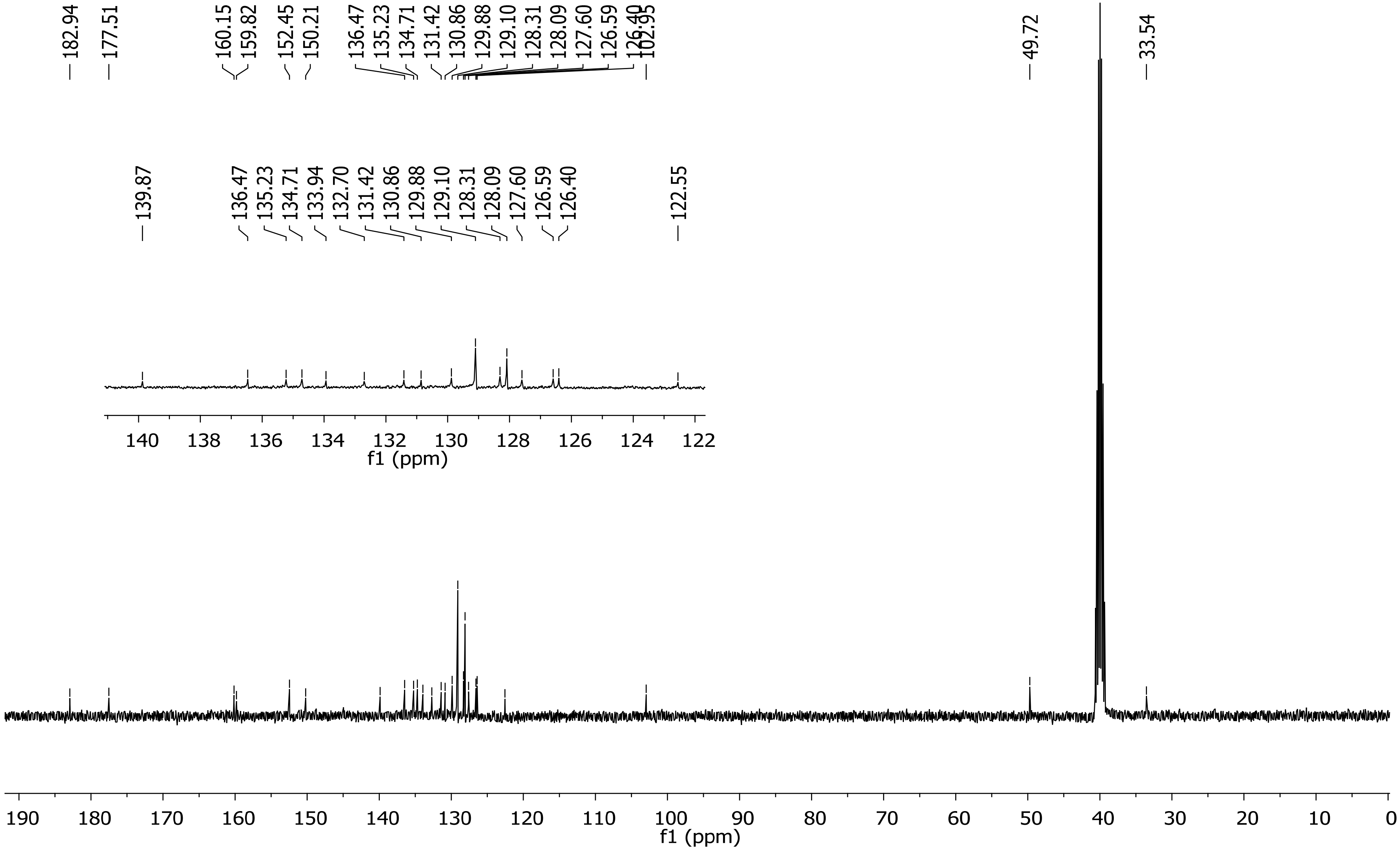

^{13}C NMR of compound **3e** (100 MHz, DMSO-d_6)

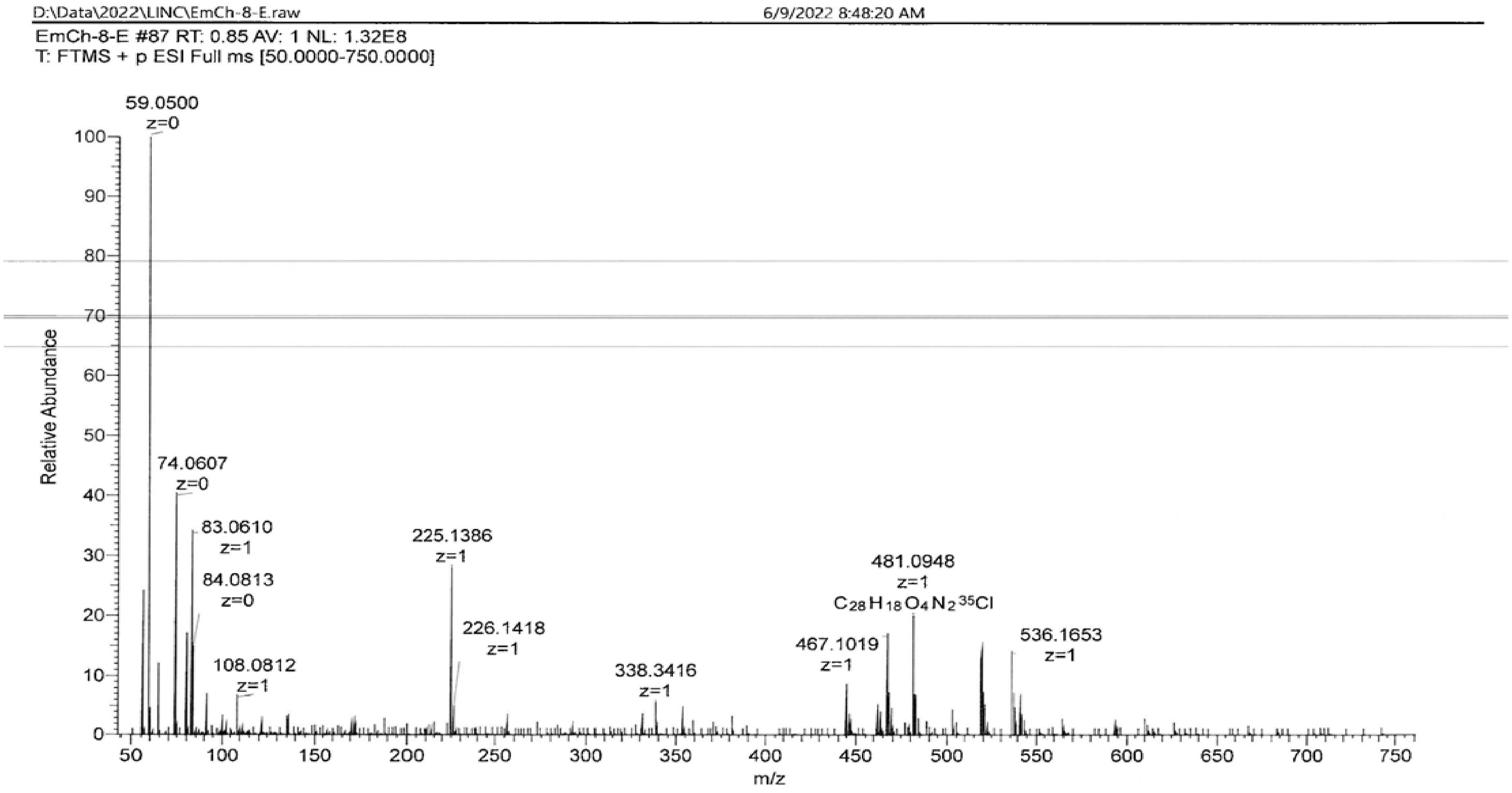

Peak Mass	Display Formula	Delta [ppm]	Theo. mass	Combined Score	Pattern Cov. [%]	MSMS Matched Fragments
481.0948	$C_{28}H_{18}O_4N_2{}^{35}Cl$	-0.30	481.09496	95.22	98.92	(Collection)

HRMS spectrum of compound **3e** (ESI, M+H$^+$)

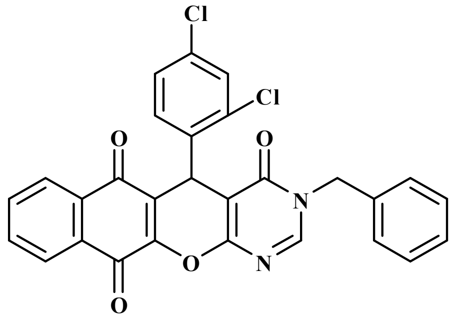

3-Benzyl-5-(2,4-dichlorophenyl)-3,5-dihydro-4*H*-benzo[6,7]chromeno[2,3-*d*]pyrimidine-4,6,11-trione (3f): It was obtained as an ochre yellow solid, yield: 37%, m.p.: > 260 °C. ^{1}H NMR (400 MHz, DMSO-*d6*) δ_H (ppm): 8.74 (s, 1H, C$\underline{H}_{pyrimidine}$), 8.08-8.06 (m, 1H), 7.88-7.85 (m, 3H), 7.49-7.44 (m, 2H), 7.33-7.25 (m, 6H), 5.46 (s, 1H, C$\underline{H}_{pyran}$), 5.11 (d, *J*= 14.7 Hz, 1H, CH_2), 4.96 (d, *J*= 14.7 Hz, 1H, CH_2). ^{13}C NMR (100 MHz, DMSO-*d6*) δ_C (ppm): 183.0, 177.4, 160.1, 159.7, 152.4, 150.2, 139.1, 136.2, 135.3, 134.9, 134.8, 133.7, 132.7, 131.3, 130.7, 129.1 (2C), 129.0, 128.3, 128.0 (2C), 127.7, 126.6, 126.4, 122.0, 102.6, 49.7, 33.1. HRMS (ESI, M+H$^+$): calcd for $C_{28}H_{17}{}^{35}Cl_2N_2O_4$; 515.0565, found; 515.0559.

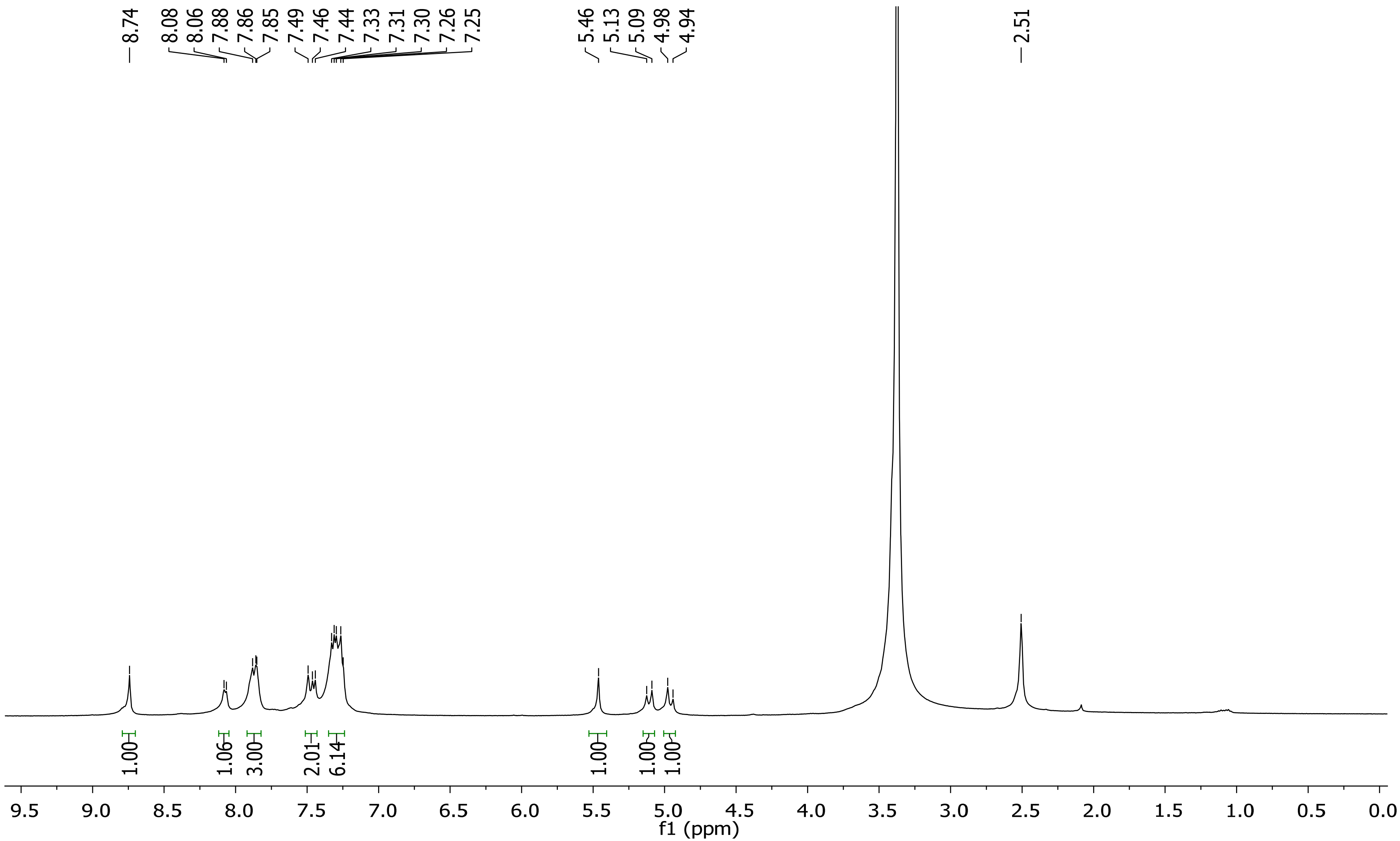

^{1}H NMR of compound **3f** (400 MHz, DMSO-d_6)

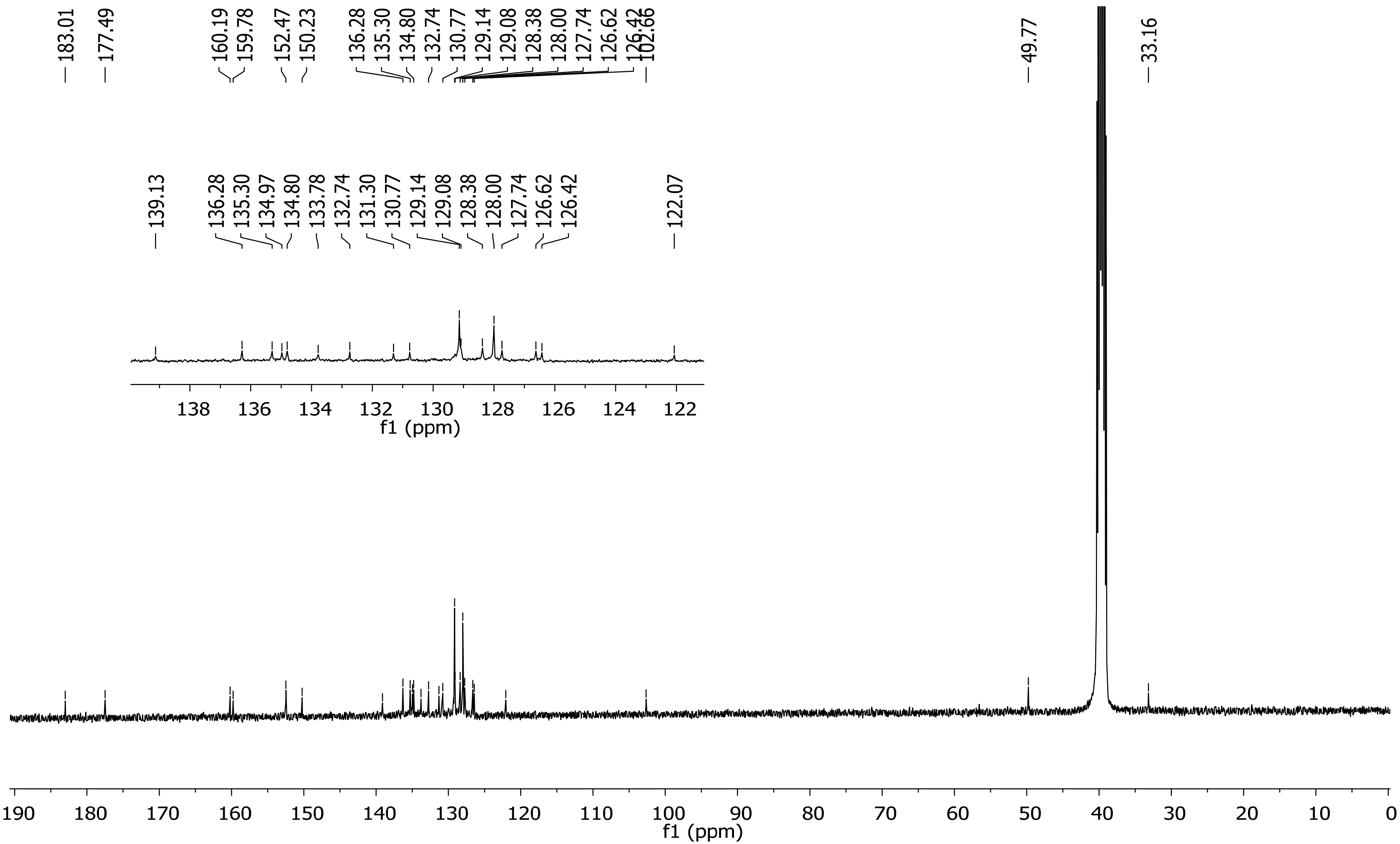

^{13}C NMR of compound **3f** (100 MHz, DMSO-d_6)

D:\Data\2022\LINC\EmCh-8-F.raw 6/9/2022 1:58:47 PM

EmCh-8-F #85 RT: 0.83 AV: 1 NL: 6.01E7
T: FTMS + p ESI Full ms [50.0000-750.0000]

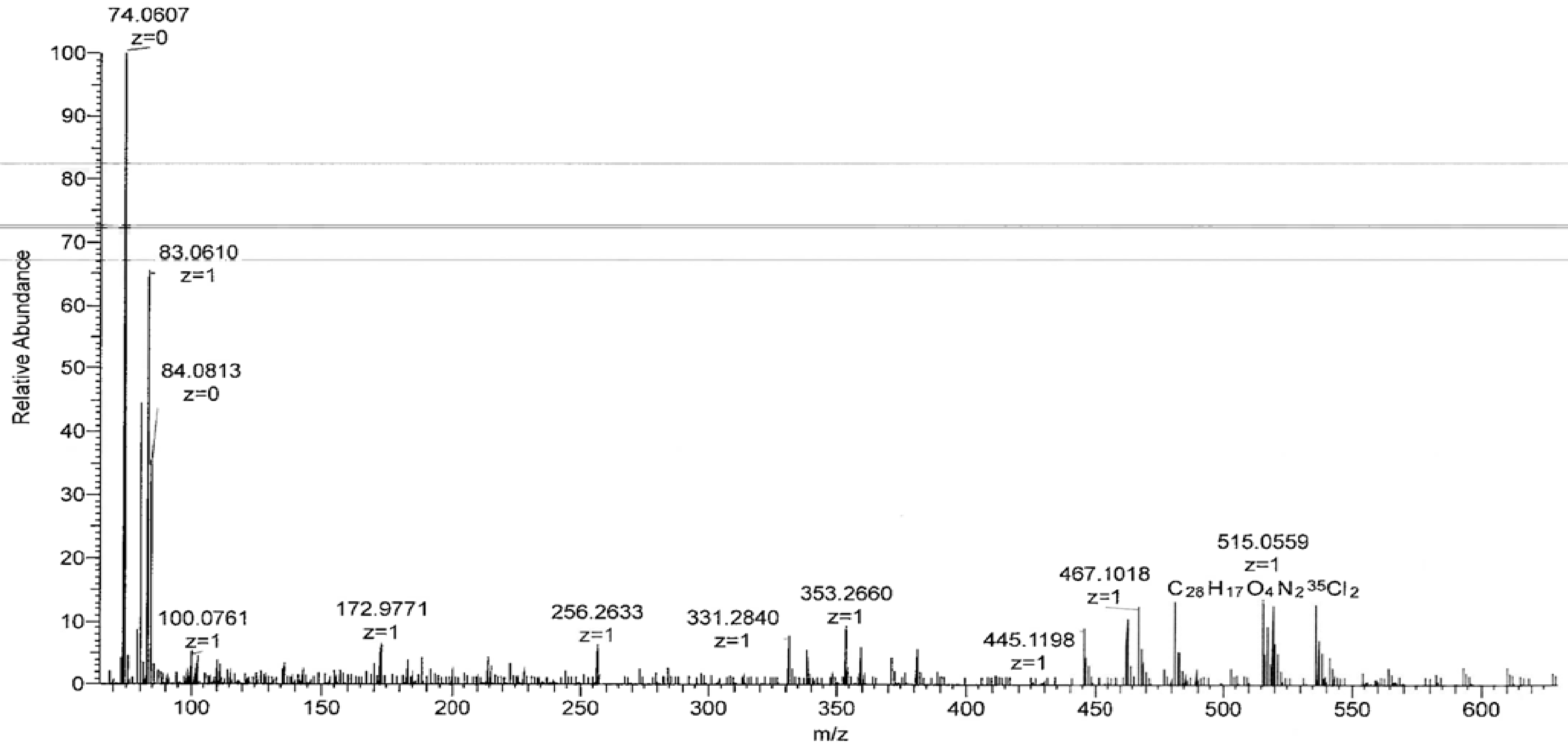

Peak Mass	Display Formula	Delta [ppm]	Theo. mass	Combined Score	Pattern Cov. [%]	MSMS Matched Fragments
515.0559	$C_{28}H_{17}O_4N_2{}^{35}Cl_2$	-0.16	515.05599	62.25	99.77	(Collection)

HRMS spectrum of compound **3f** (ESI, M+H$^+$)

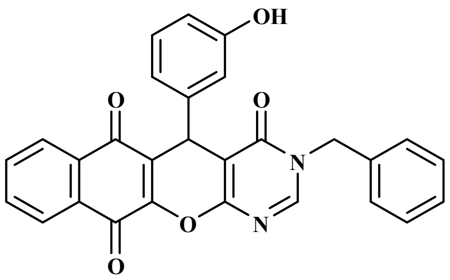

3-Benzyl-5-(3-hydroxyphenyl)-3,5-dihydro-4*H*-benzo[6,7]chromeno[2,3-*d*]pyrimidine-4,6,11-trione (3g): It was obtained as a red solid brick, yield: 57%, m.p.: 200-220 °C. ^{1}H NMR (400 MHz, DMSO-*d6*) δ_H (ppm): 9.37 (s, 1H, OH), 8.74 (s, 1H, C$\underline{H}_{pyrimidine}$), 8.07 (m, 1H), 7.93-7.85 (m, 3H), 7.34-7.31 (m, 5H), 7.05-7.02 (m, 1H), 6.80-6.76 (m, 2H), 6.56 (d, *J*= 7.9 Hz, 1H), 5.15 (d, *J*= 14.6 Hz, 1H, CH_2), 5.04 (s, 1H, C$\underline{H}_{pyran}$), 5.00 (d, *J*= 14.6 Hz, 1H, CH_2). ^{13}C NMR (100 MHz, DMSO-*d6*) δ_C (ppm): 183.0, 177.5, 160.4, 159.7, 157.6, 152.1, 149.9, 143.6, 136.4, 135.1, 134.7, 131.4, 131.0, 129.7, 129.1 (2C), 128.3, 128.2 (2C), 126.6, 126.4, 123.5, 119.7, 116.1, 114.6, 103.7, 49.8, 34.5. HRMS (ESI, M+H$^+$): calcd for $C_{28}H_{19}N_2O_5$; 463.1294, found; 463.1286.

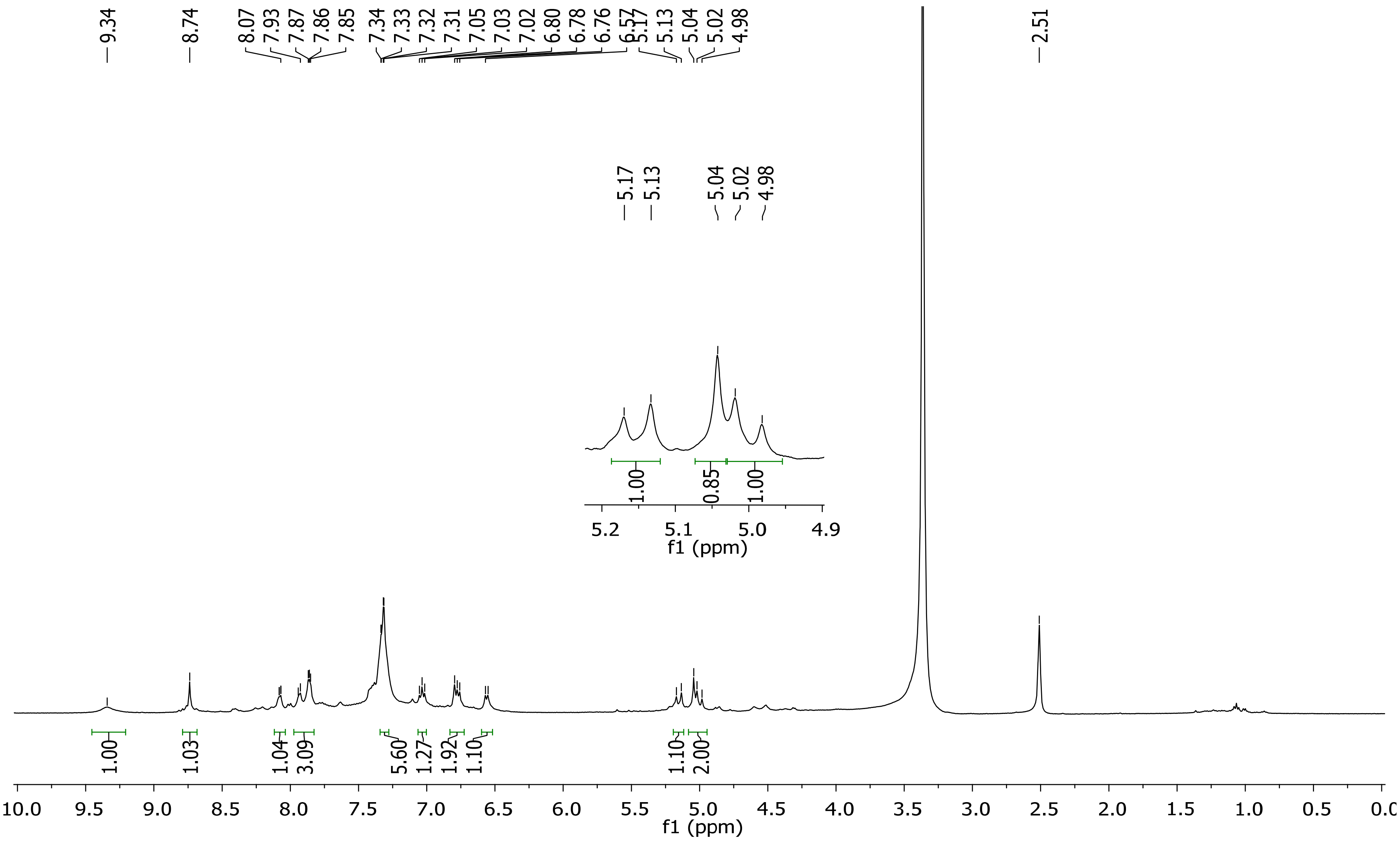

^{1}H NMR of compound **3g** (400 MHz, DMSO-d_6)

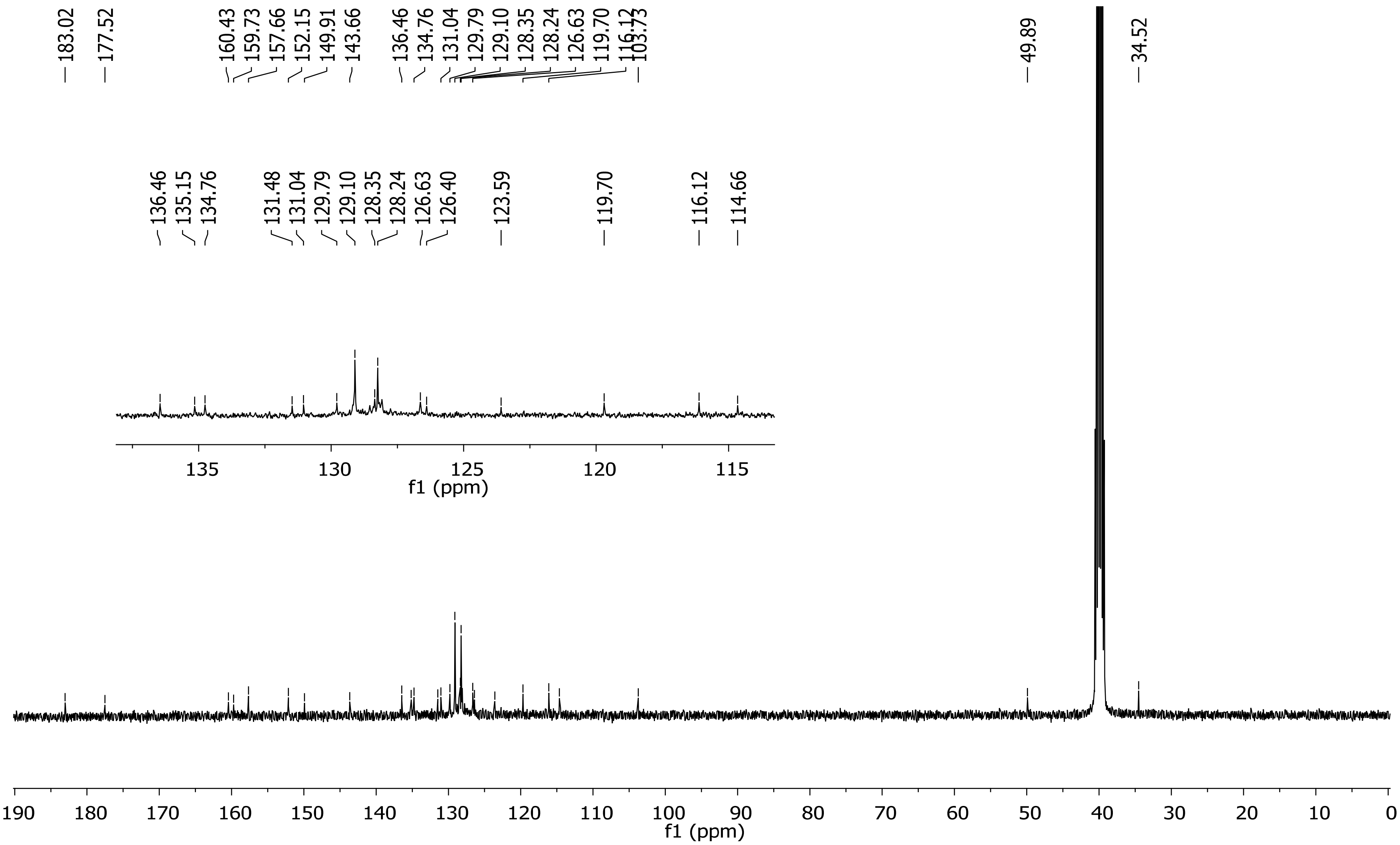

^{13}C NMR of compound **3g** (100 MHz, DMSO-d_6)

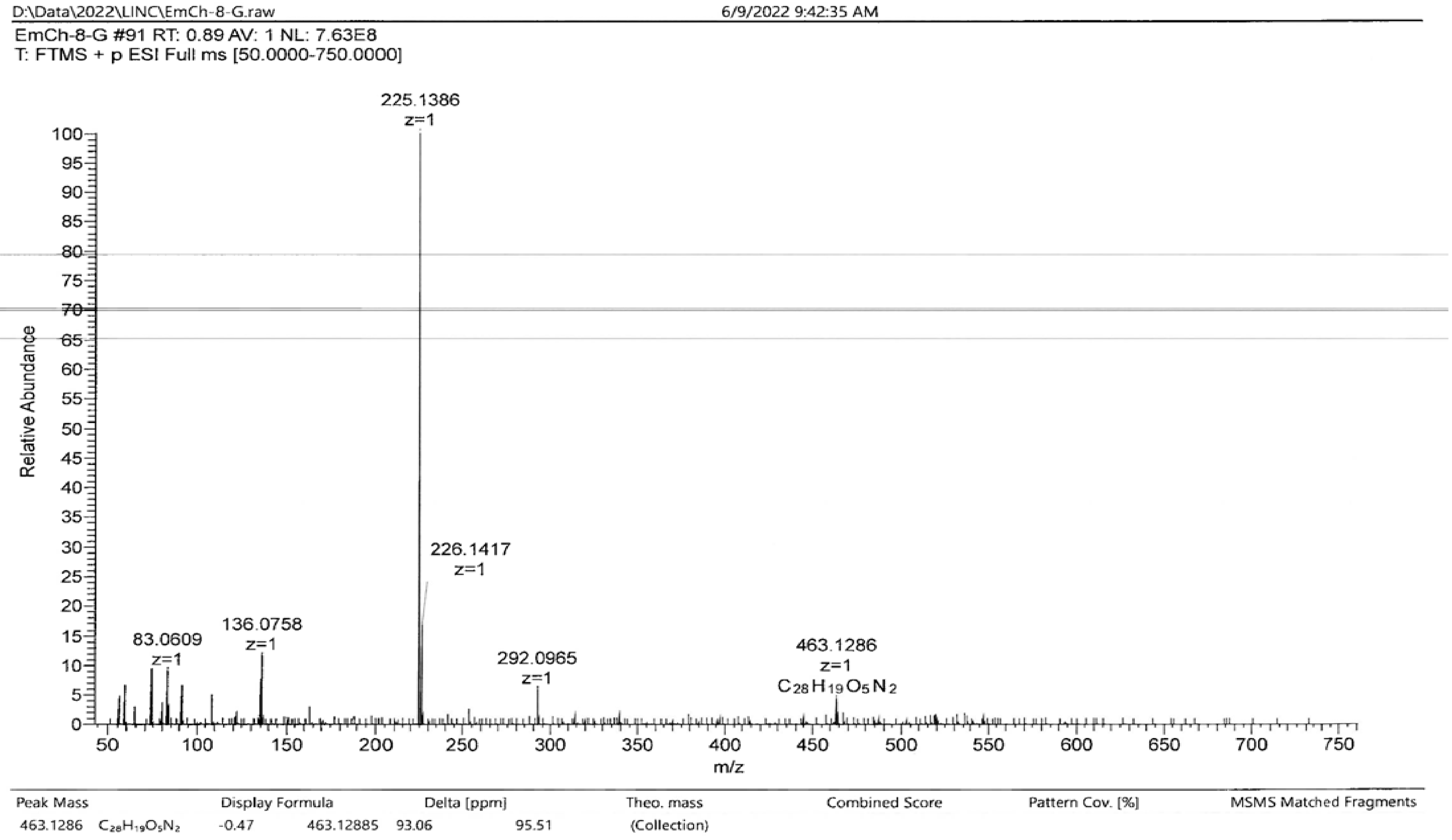

HRMS spectrum of compound **3g** (ESI, $M+H^+$)

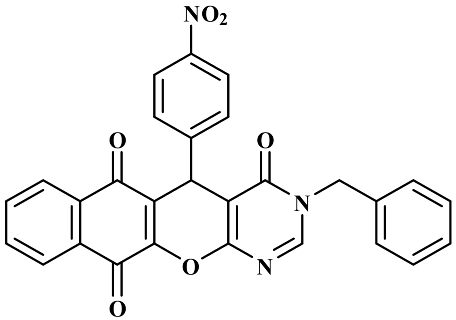

3-Benzyl-5-(4-nitrophenyl)-3,5-dihydro-4*H*-benzo[6,7]chromeno[2,3-*d*]pyrimidine-4,6,11-trione (3h): It was obtained as an ochre yellow solid, yield: 50%, m.p.: > 260 °C. ^{1}H NMR (400 MHz, DMSO-*d6*) δ_H (ppm): 8.73 (s, 1H, C$\underline{H}_{pyrimidine}$), 8.10-8.08 (m, 3H), 7.87-7.81 (m, 3H), 7.70 (d, *J*= 8.5 Hz, 2H), 7.32-7.27 (m, 5H), 5.21 (s, 1H, C$\underline{H}_{pyran}$), 5.09 (d, *J*= 14.7 Hz, 1H, CH_2), 5.47 (d, *J*= 14.7 Hz, 1H, CH_2). ^{13}C NMR (100 MHz, DMSO-*d6*) δ_C (ppm): 183.0, 177.3, 160.4, 159.7, 152.6, 150.4, 149.7, 146.9, 136.2, 135.1, 134.8, 131.3, 131.0, 130.8 (2C), 129.1 (2C), 128.4, 128.2 (2C), 126.6, 126.3, 123.7 (2C), 122.0, 102.6, 49.9, 35.3. HRMS (ESI, M+H$^+$): calcd for $C_{28}H_{18}N_3O_6$; 492.1196, found; 492.1192.

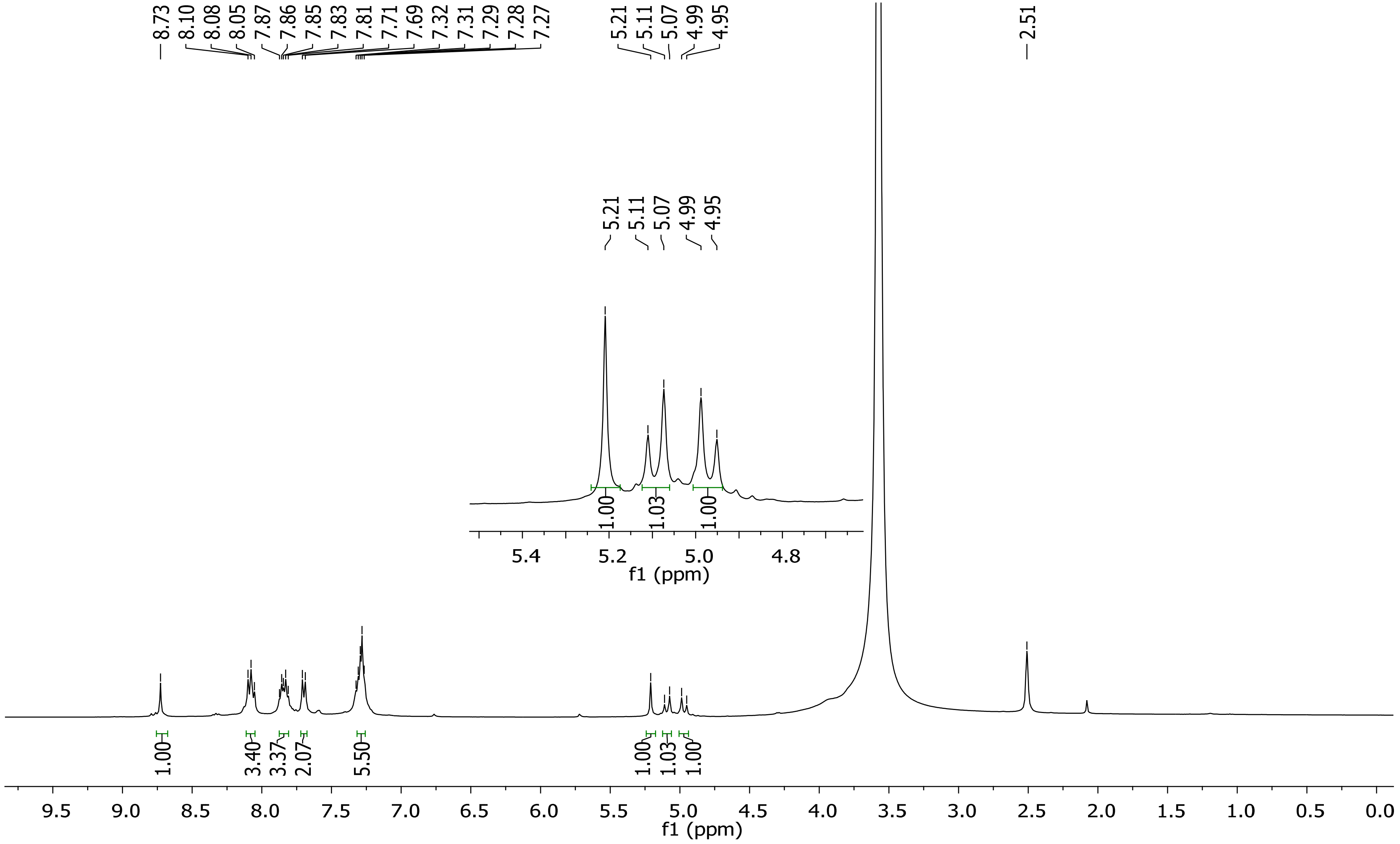

^{1}H NMR of compound **3h** (400 MHz, DMSO-d_6)

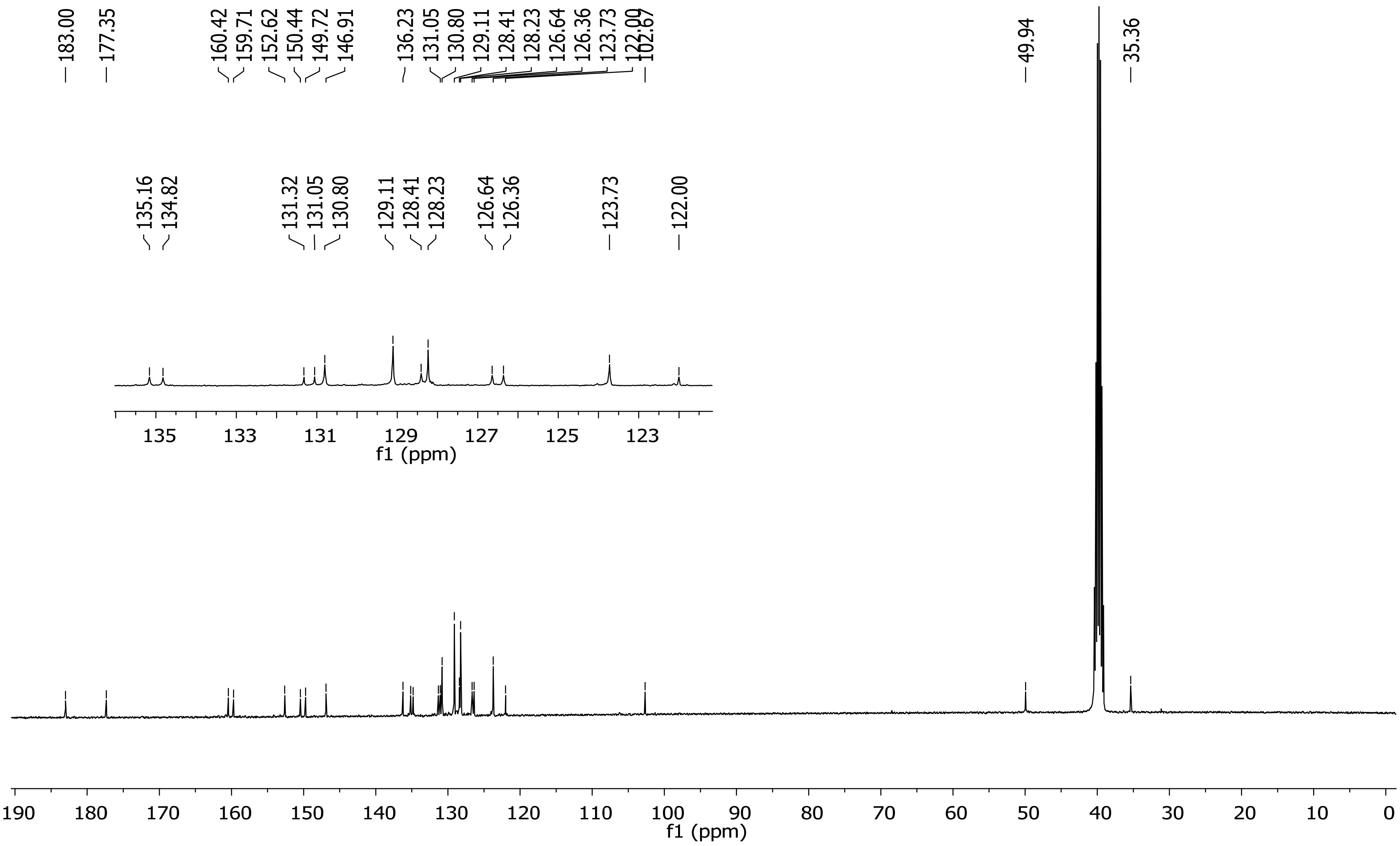

^{13}C NMR of compound **3h** (100 MHz, DMSO-d_6)

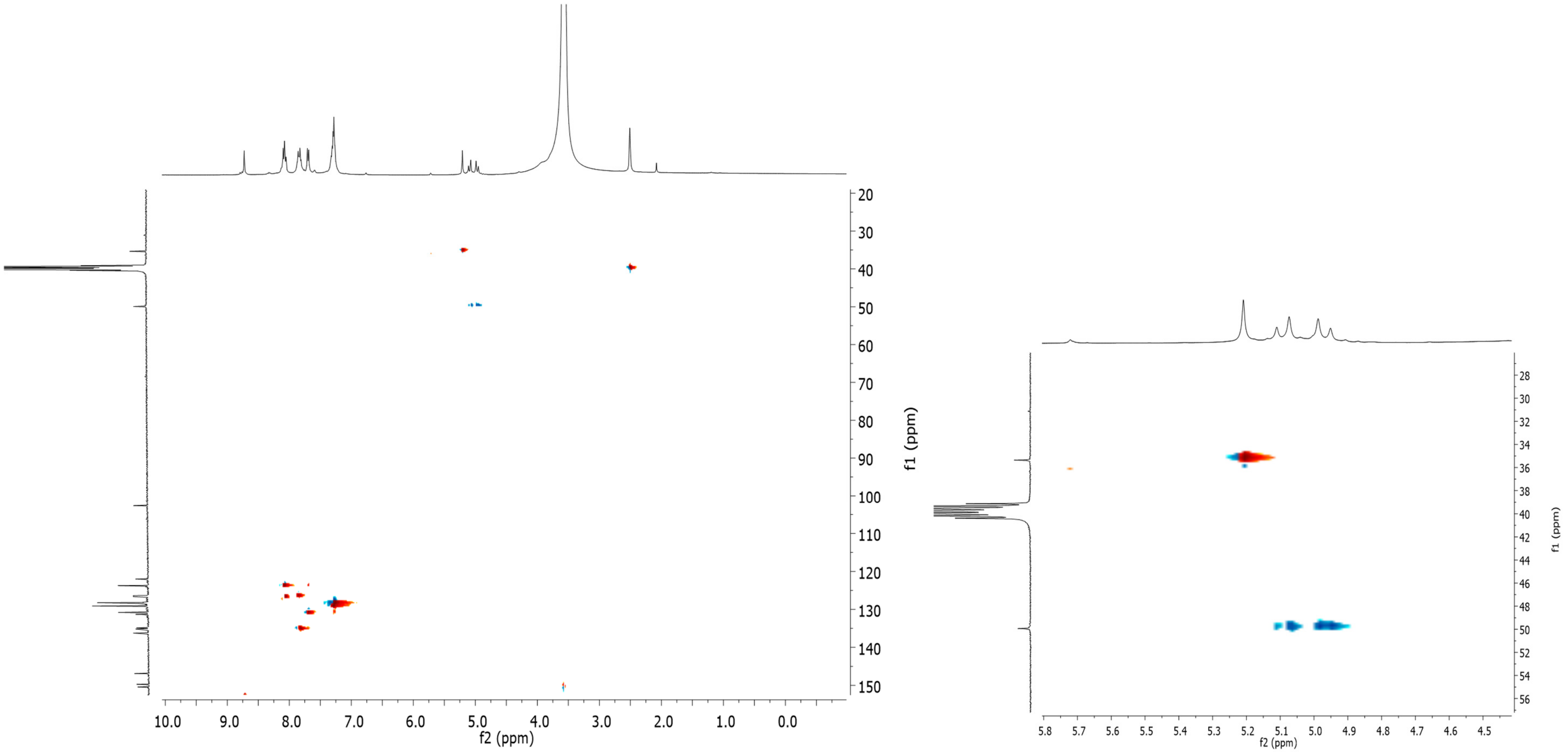

2D HSQC spectrum of compound **3h**

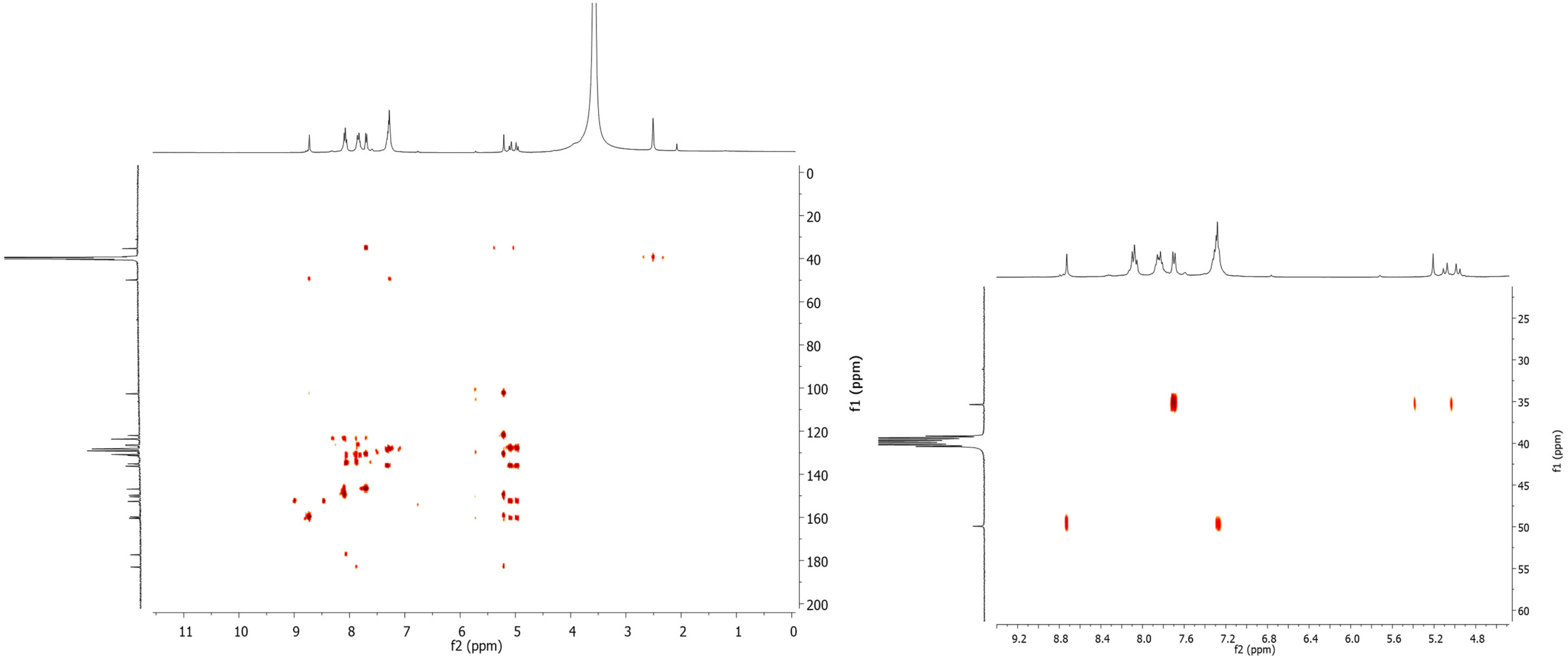

2D HMBC spectrum of compound **3h**

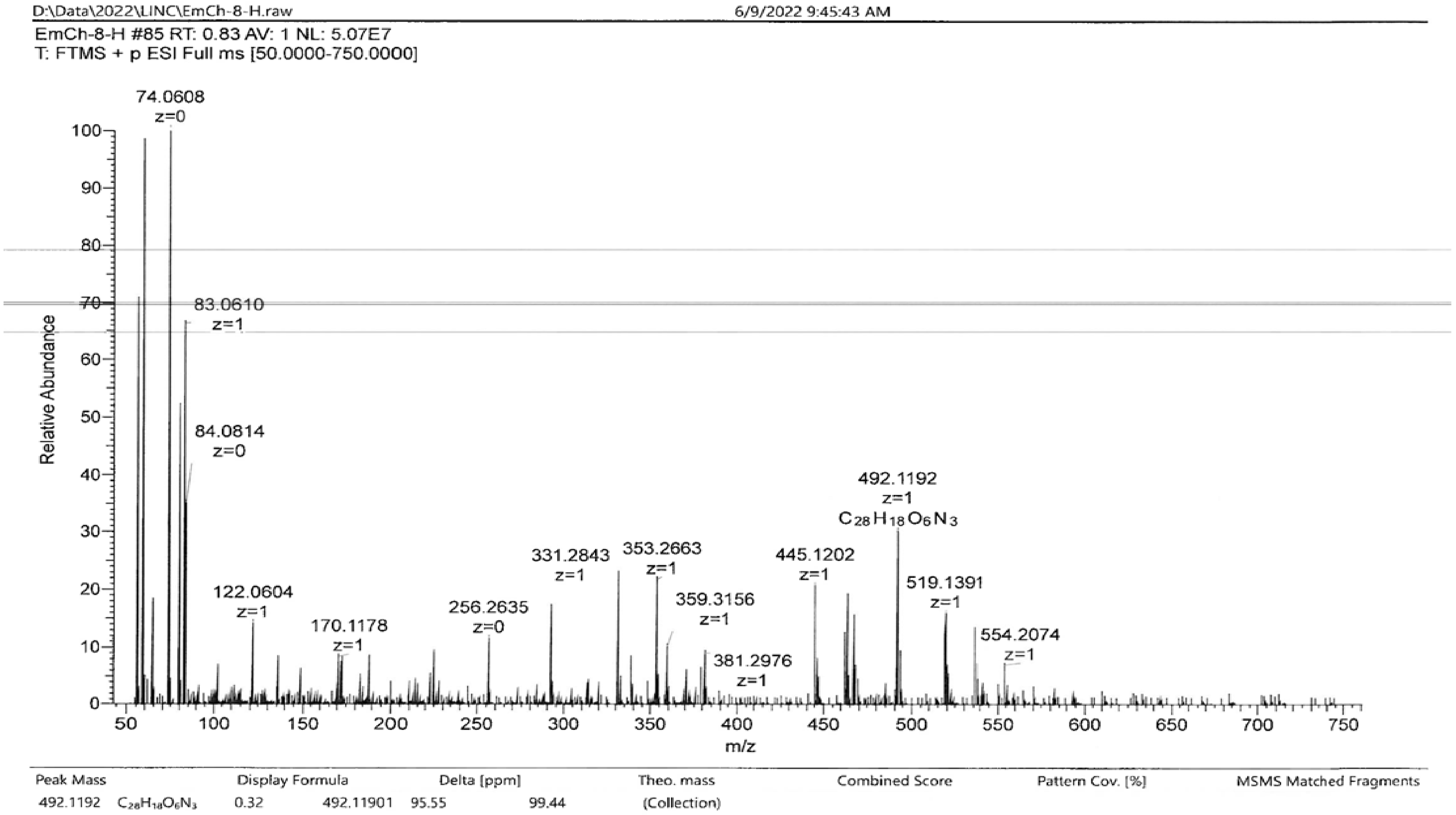

HRMS spectrum of compound **3h** (ESI, M+H$^+$)

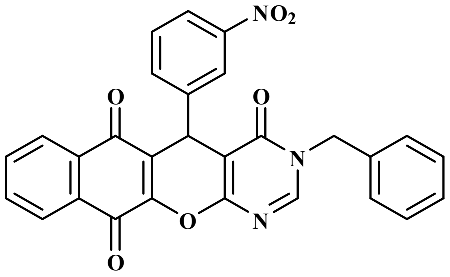

3-Benzyl-5-(3-nitrophenyl)-3,5-dihydro-4*H*-benzo[6,7]chromeno[2,3-*d*]pyrimidine-4,6,11-trione (3i): It was obtained as an orange solid, yield: 52%, m.p.: 258-260 °C. ^{1}H NMR (400 MHz, DMSO-*d6*) δ_H (ppm): 8.76 (s, 1H, C$\underline{H}_{pyrimidine}$), 8.24 (s, 1H), 8.06-8.04 (m, 2H), 7.89-7.85 (m, 4H), 7.56 (m, 1H), 7.29 (m, 5H), 5.25 (s, 1H, C$\underline{H}_{pyran}$), 5.12 (d, *J*= 14.7 Hz, 1H, CH_2), 4.98 (d, *J*= 14.7 Hz, 1H, CH_2). ^{13}C NMR (100 MHz, DMSO-*d6*) δ_C (ppm): 183.0, 177.3, 160.4, 159.7, 152.7, 150.5, 148.0, 144.4, 136.3, 136.1, 135.0, 134.7, 131.4, 131.2, 130.1, 129.0 (2C), 128.3, 128.2 (2C), 126.6, 126.3, 124.0, 122.6, 121.8, 102.8, 49.9, 35.2. HRMS (ESI, M+H$^+$): calcd for $C_{28}H_{18}N_3O_6$; 492.1196, found; 492.1190.

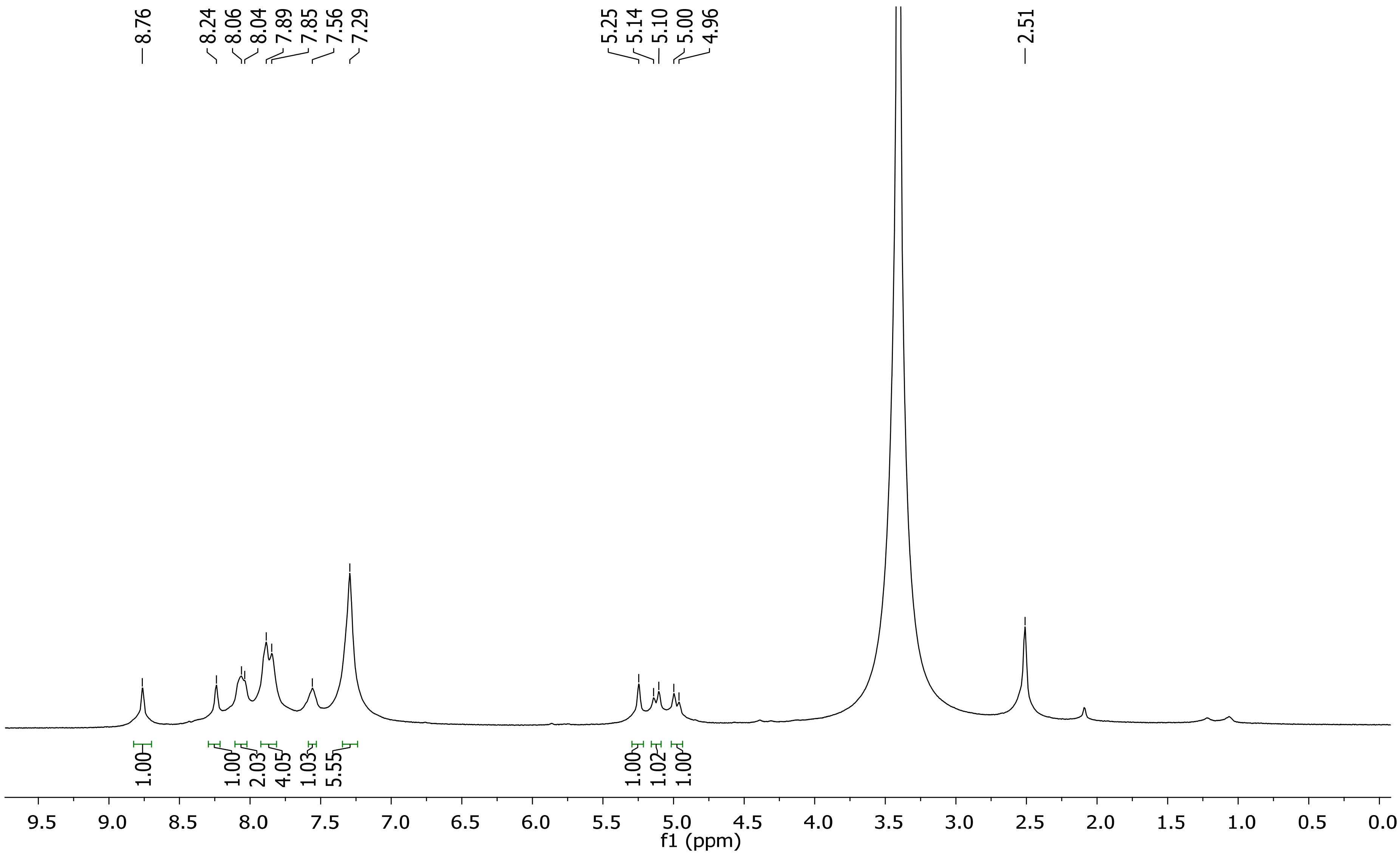

^{1}H NMR of compound **3i** (400 MHz, DMSO-d_6)

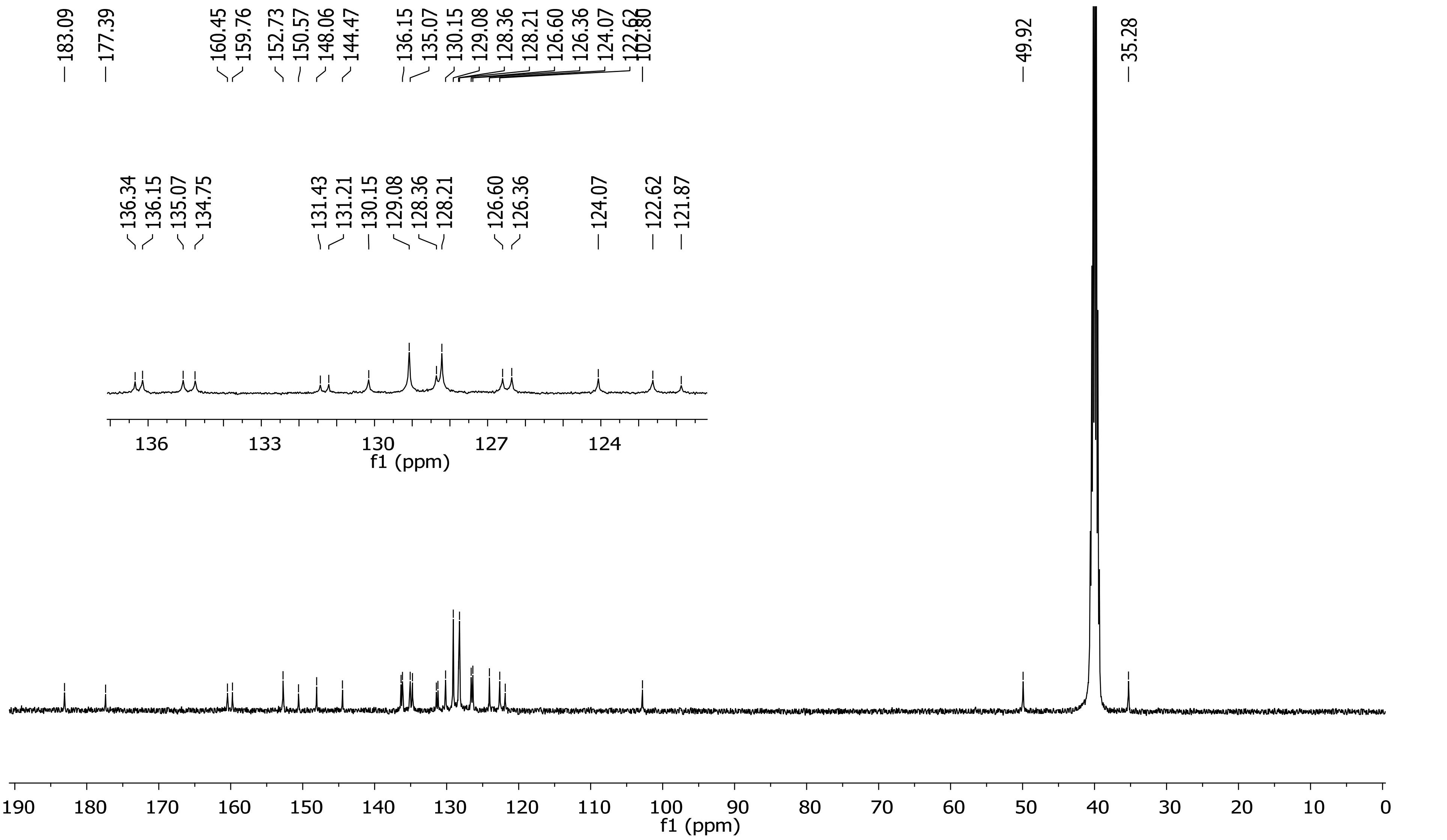

^{13}C NMR of compound **3i** (100 MHz, DMSO-d_6)

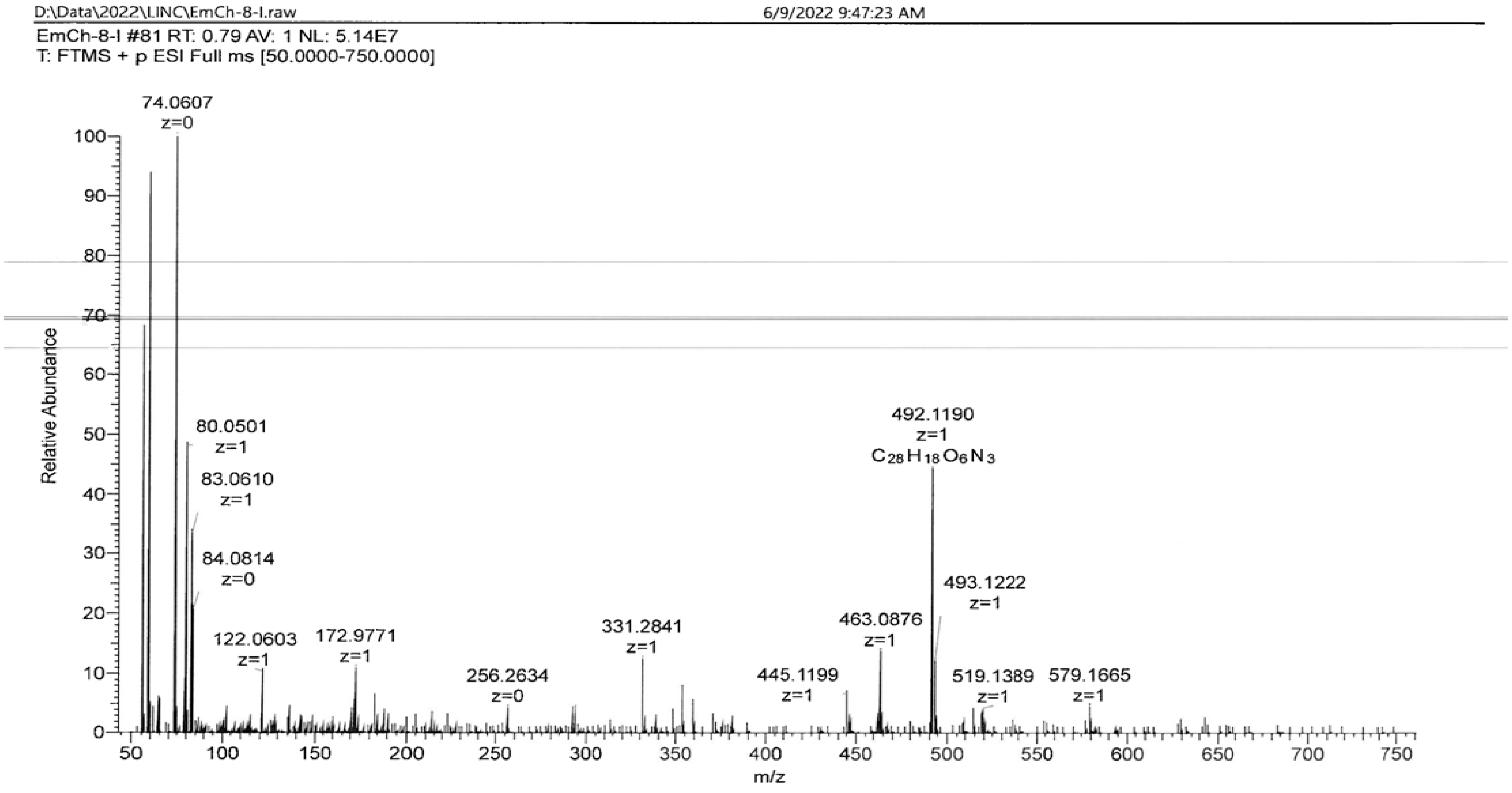

Peak Mass	Display Formula	Delta [ppm]	Theo. mass	Combined Score	Pattern Cov. [%]	MSMS Matched Fragments
492.1190	$C_{28}H_{18}O_6N_3$	-0.05	492.11901	98.22	100	(Collection)

HRMS spectrum of compound **3i** (ESI, M+H$^+$)

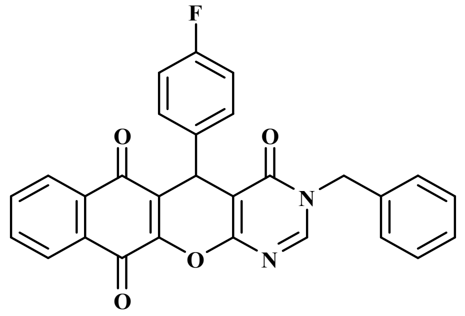

3-Benzyl-5-(4-fluorophenyl)-3,5-dihydro-4*H*-benzo[6,7]chromeno[2,3-*d*]pyrimidine-4,6,11-trione (3j): It was obtained as an ochre yellow solid, yield: 51%, m.p.: > 260 °C. ^{1}H NMR (400 MHz, DMSO-*d6*) δ_H (ppm): 8.73 (s, 1H, C$\underline{H}_{pyrimidine}$), 8.09-8.07 (m, 1H), 7.93-7.83 (m, 3H), 7.45-7.42 (m, 2H), 7.33-7.26 (m, 5H), 7.09-7.05 (m, 2H), 5.13 (d, *J*= 14.7 Hz, 1H, CH_2), 5.12 (s, 1H, C$\underline{H}_{pyran}$), 4.99 (d, *J*= 14.7 Hz, 1H, CH_2). ^{13}C NMR (100 MHz, DMSO-*d6*) δ_C (ppm): 183.1, 177.4, 160.4, 159.6, 152.3, 150.1, 138.6, 136.4, 135.0, 134.7, 131.4 (2C), 131.2, 131.1, 129.0 (2C), 128.3, 128.2 (2C), 126.6, 126.3, 122.9, 115.5, 115.3, 103.5, 49.8, 34.2. HRMS (ESI, M+H$^+$): calcd for $C_{28}H_{18}FN_2O_4$; 465.1251, found; 465.1244.

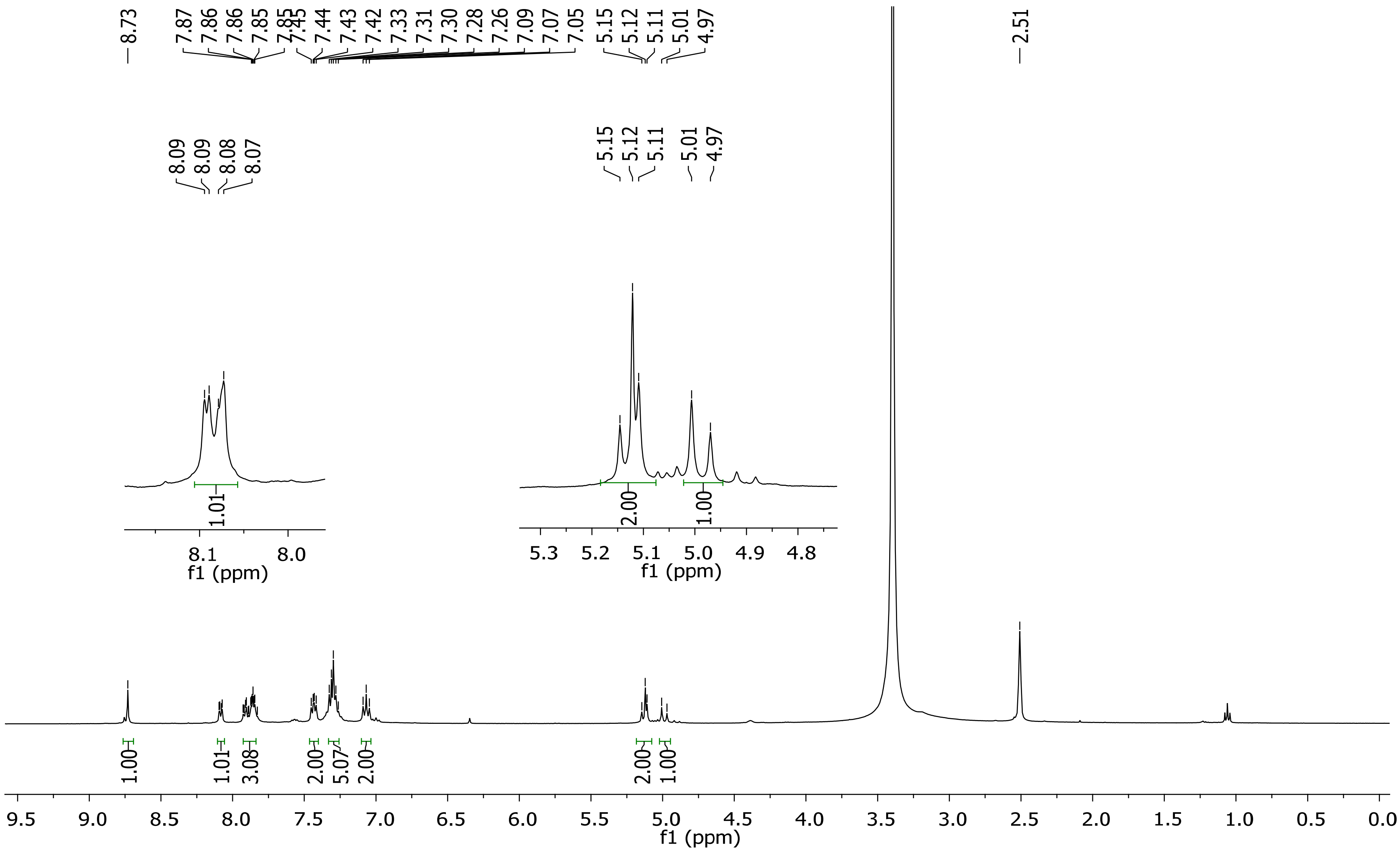

^{1}H NMR of compound **3j** (400 MHz, DMSO-d_6)

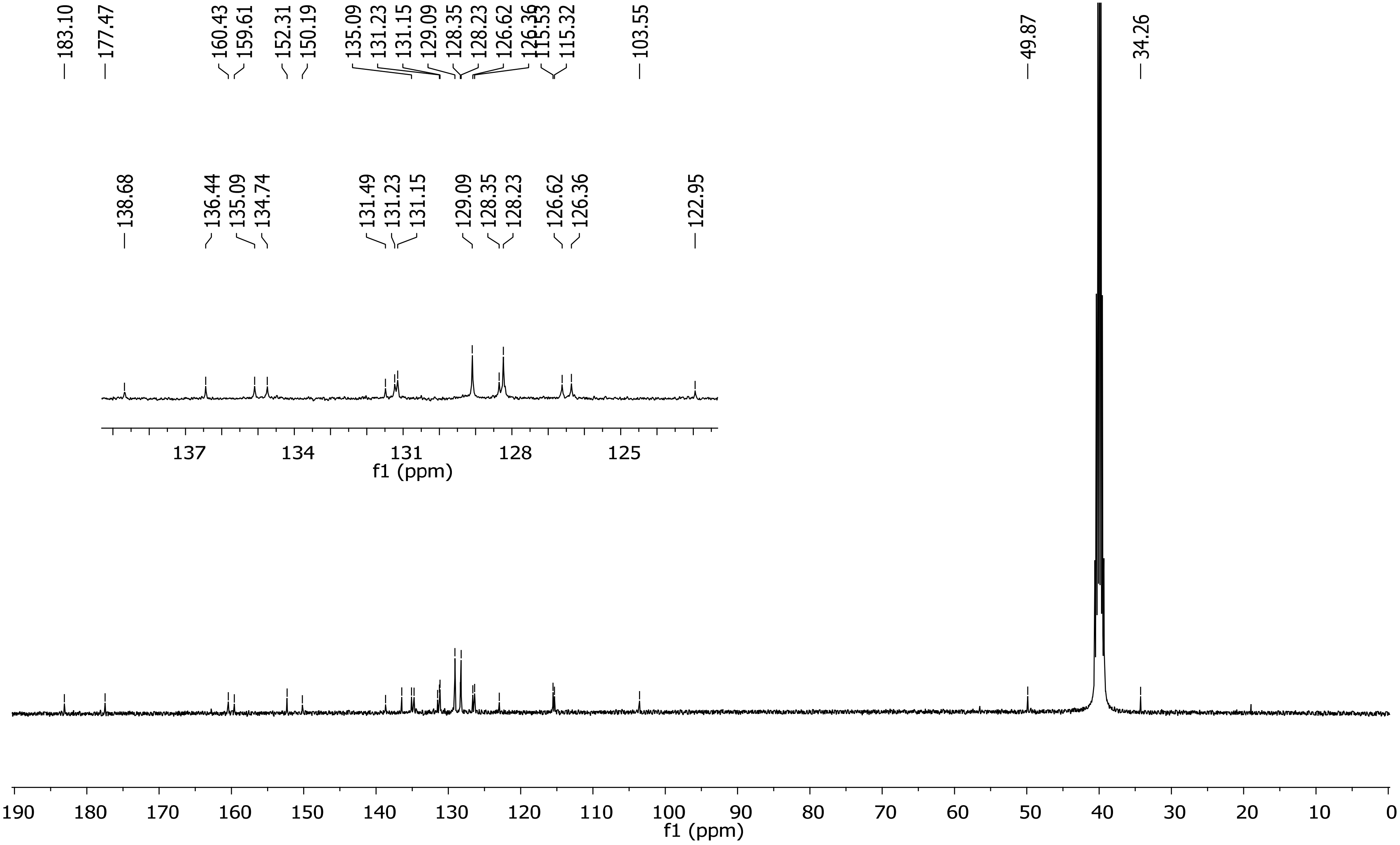

^{13}C NMR of compound **3j** (100 MHz, DMSO-*d*$_6$)

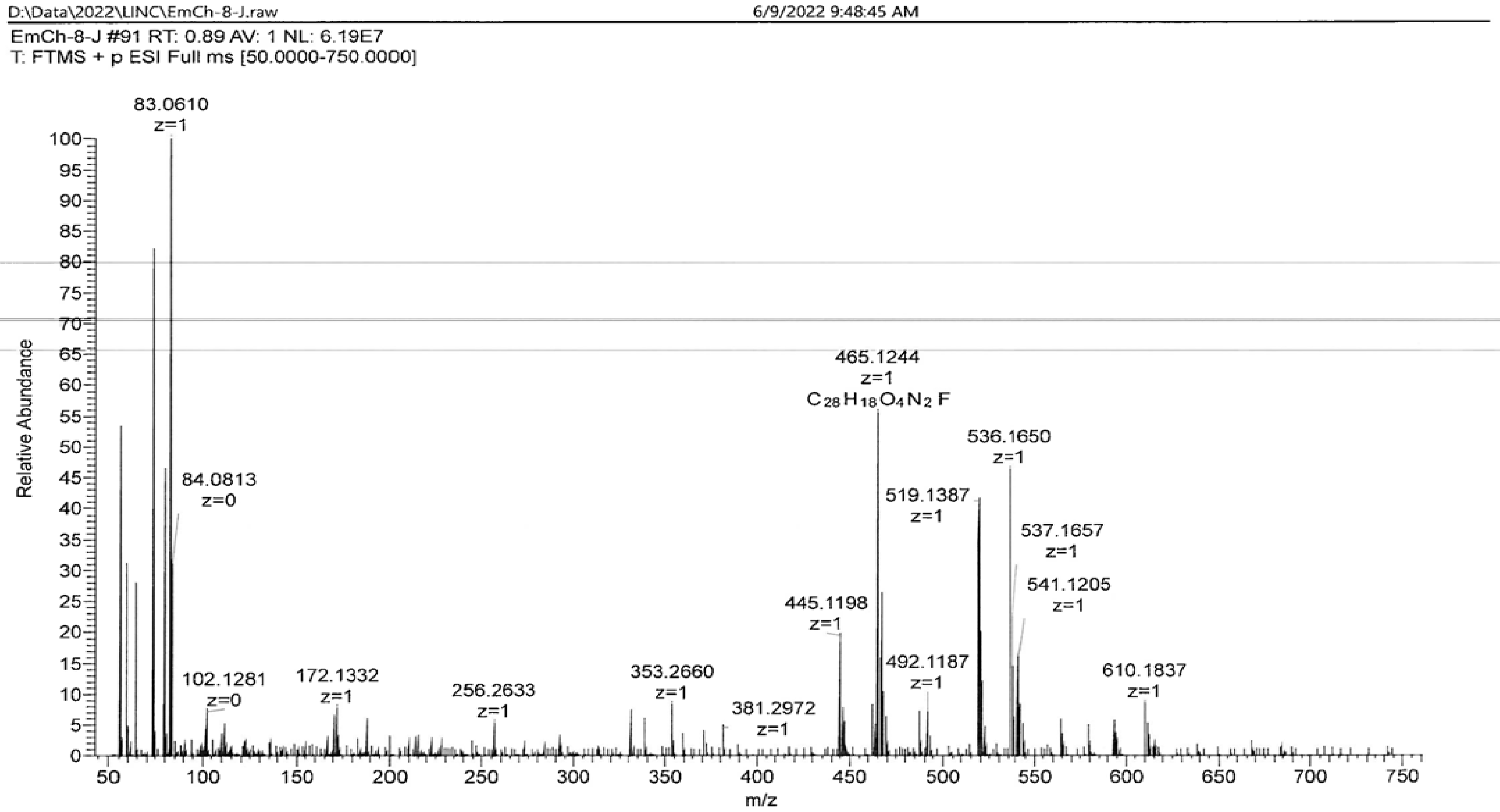

Peak Mass	Display Formula	Delta [ppm]	Theo. mass	Combined Score	Pattern Cov. [%]	MSMS Matched Fragments
465.1244	$C_{28}H_{18}O_4N_2F$	-0.26	465.12451	66.66	100	(Collection)

HRMS spectrum of compound **3j** (ESI, $M+H^+$)
